# A systematic evidence map for the evaluation of noncancer health effects and exposures to polychlorinated biphenyl mixtures

**DOI:** 10.1016/j.envres.2022.115148

**Published:** 2022-12-26

**Authors:** Laura M. Carlson, Krista Christensen, Sharon K. Sagiv, Pradeep Rajan, Carolyn R. Klocke, Pamela J. Lein, Evan Coffman, Rachel M. Shaffer, Erin E. Yost, Xabier Arzuaga, Pam Factor-Litvak, Alexander Sergeev, Michal Toborek, Michael S. Bloom, Joanne Trgovcich, Todd A. Jusko, Larry Robertson, John D. Meeker, Aileen F. Keating, Robyn Blain, Raquel A. Silva, Samantha Snow, Cynthia Lin, Kelly Shipkowski, Brandall Ingle, Geniece M. Lehmann

**Affiliations:** a Office of Research and Development, Center for Public Health and Environmental Assessment, US Environmental Protection Agency, USA; b Center for Environmental Research and Children’s Health (CERCH), School of Public Health, University of California, Berkeley, CA, USA; c Pradeep Rajan LLC, Chapel Hill, NC, USA; d Department of Molecular Biosciences, University of California, Davis School of Veterinary Medicine, Davis, CA, USA; e Mailman School of Public Health, Columbia University, New York, NY, USA; f Ohio University, Athens, OH, USA; g University of Miami, Miami, FL, USA; h George Mason University, Fairfax, VA, USA; i ICF, Fairfax, VA, USA; j University of Rochester School of Medicine and Dentistry, Rochester, NY, USA; k University of Iowa, Iowa City, IA, USA; l University of Michigan, Ann Arbor, MI, USA; m Department of Animal Science, Iowa State University, Ames, IA, USA; n ICF, Fairfax, VA, Currently at National Institute of Environmental Health Sciences, USA; o ICF, Fairfax, VA, Currently at US Environmental Protection Agency, USA

**Keywords:** Evidence map, Polychlorinated biphenyl, Systematic review, Risk assessment, Hazard identification

## Abstract

Assessing health outcomes associated with exposure to polychlorinated biphenyls (PCBs) is important given their persistent and ubiquitous nature. PCBs are classified as a Group 1 carcinogen, but the full range of potential noncancer health effects from exposure to PCBs has not been systematically summarized and evaluated. We used systematic review methods to identify and screen the literature using combined manual review and machine learning approaches. A protocol was developed that describes the literature search strategy and Populations, Exposures, Comparators, and Outcomes (PECO) criteria used to facilitate subsequent screening and categorization of literature into a systematic evidence map of PCB exposure and noncancer health endpoints across 15 organs/systems. A comprehensive literature search yielded 62,599 records. After electronic prioritization steps, 17,037 studies were manually screened at the title and abstract level. An additional 900 studies identified by experts or supplemental searches were also included. After full-text screening of 3889 references, 1586 studies met the PECO criteria. Relevant study details such as the endpoints assessed, exposure duration, and species were extracted into literature summary tables. This review compiles and organizes the human and mammalian studies from these tables into an evidence map for noncancer health endpoints and PCB mixture exposure to identify areas of robust research as well as areas of uncertainty that would benefit from future investigation. Summary data are available online as interactive visuals with downloadable metadata. Sufficient research is available to inform PCB hazard assessments for most organs/systems, but the amount of data to inform associations with specific endpoints differs. Furthermore, despite many years of research, sparse data exist for inhalation and dermal exposures, which are highly relevant human exposure routes. This evidence map provides a foundation for future systematic reviews and noncancer hazard assessments of PCB mixtures and for strategic planning of research to inform areas of greater uncertainty.

## 1. Introduction

Polychlorinated biphenyls (PCBs) are halogenated organic pollutants consisting of 209 congeners varying in the number and position of chlorine atoms substituted on biphenyl rings. PCBs were produced as technical mixtures (e.g., Aroclors) containing numerous individual congeners. Mixtures of these congeners were used as dielectric fluids in capacitors and transformers, as lubricants, and as additives in a variety of other products, including paints and caulk. PCB production was banned in the United States in 1979 (US EPA, 1979) and in most of the world by the time the Stockholm Convention was adopted in 2001 (SCPOP, 2008). Even so, because of their widespread use, disposal, and resistance to degradation, these chemicals are pervasive in the environment and biota.

Humans are exposed to PCBs throughout their lifetimes, including prenatally and during the early postnatal period through breastfeeding (van den Berg et al., 2017), with continuing exposure through multiple routes including diet and inhalation (Weitekamp et al., 2021). Occupational exposure historically occurred during production or use of PCBs and PCB containing products (Wolff, 1985), and may still occur, for example through maintenance, repair, or recycling of old PCB containing products or disturbance of construction materials containing PCBs (Okeme and Arrandale, 2019; Herrick et al., 2007). The general population can be exposed to PCBs by ingesting contaminated food (especially fish from contaminated waters), and by inhaling contaminated air, in both indoor and outdoor settings, especially at locations which still use electrical equipment and/or building and construction products containing PCBs. The issue of potential inhalation exposure to PCBs in contaminated buildings, including some schools, is an environmental health topic that has received much attention from the US Environmental Protection Agency (US EPA, 2019, 2015, 2012) and the media (e.g., Associated Press, 2022; Blatchhford, 2022).

The PCB mixtures associated with each exposure pathway differ from each other and from the original technical mixtures due to differential degradation of the individual congeners in the environment over time, variable partitioning of PCB congeners in environmental compartments, and differences in toxicokinetics. Another factor contributing to differences between modern environmental PCB mixtures and technical mixtures is the ongoing, inadvertent PCB production that occurs during certain manufacturing processes, such as pigment production (Zhao et al., 2020a; Vorkamp, 2015; Hu and Hornbuckle, 2010). Therefore, humans are exposed to different environmental mixtures of PCBs from different sources (Ampleman et al., 2015; Hornbuckle and Robertson, 2010) that have distinct congener compositions from the original produced mixtures. Furthermore, the composition of an exogenous environmental PCB mixture is subject to change after uptake into the human body as different congeners are metabolized and eliminated at different rates. Consequently, relating measures of PCB congeners in biological matrices to their corresponding environmental source mixtures can be challenging, which complicates traditional risk assessment (Christensen et al., 2021).

Associations between PCB exposure and cancer and noncancer health endpoints have been reported in both human epidemiological and experimental animal studies (IARC, 2015; ATSDR, 2000). Of the 209 congeners, approximately 12 are considered “dioxin like,” meaning that they bind to the aryl hydrocarbon receptor (AhR) and can interact with biological systems through the same mode of action as 2,3,7,8-tetrachlorodibenzo-p-dioxin (van den Berg et al., 2006; Poland et al., 1976). The remaining “nondioxin-like” congeners have also been reported to be associated with health endpoints; however, the specific hazards, dose-response relationships, and modes of action are variable and not as clearly understood for these PCBs. National and international health agencies have assessed PCB toxicity, including cancer (IARC, 2015; US EPA, 1996) and noncancer endpoints (ATSDR, 2000; US EPA, 1994, 1993). The International Agency for Research on Cancer (IARC) has classified PCBs as Group 1 carcinogens (IARC, 2015). However, the most comprehensive review of the potential effects of PCB exposure to date was conducted by the Agency for Toxic Substances and Disease Registry (ATSDR) (ATSDR, 2011, 2000). These reviews identified several noncancer outcomes sensitive to PCB exposure, including dermal, ocular, immune, thyroid, liver, reproductive, developmental, and neurodevelopmental effects (ATSDR, 2000). New research since ATSDR’s assessments suggests the potential for additional health hazards, notably metabolic and cardiovascular effects. Furthermore, the full database of PCB literature has not previously been reviewed using systematic methods with the intent to identify most of the available data informing potential noncancer health hazards of exposure to PCB mixtures.

Evidence mapping is a useful tool to gain appreciation for the size and content of a literature database. Systematic evidence maps are used as analysis tools, which “do not seek to synthesize evidence but instead to catalog it, utilizing systematic search and selection strategies to produce searchable databases of studies along with detailed descriptive information” (Elsevier, 2017). In this approach, systematic review methods are used, including a targeted search of the literature guided by Populations, Exposures, Comparators, and Outcomes (PECO) criteria (Table 1) and subsequent study categorization and development of visualizations to “map” the contents of the database. The resulting map can be used to evaluate the data available to inform specific questions that could be of interest for future systematic reviews.

This evidence map’s main objective is to assemble and summarize a previously uncharacterized literature base of noncancer health outcome data collected alongside information on exposures to PCB mixtures. By identifying health endpoints with databases sufficient to support evaluations of coherence across evidence streams (i.e., from studies in humans and nonhuman mammals) and across biologically related endpoints (e.g., endpoints linked through a common adverse outcome pathway), we can highlight the databases with the highest likelihood of supporting an analysis of causal relationships with exposure to PCB mixtures for future systematic reviews. Conversely, by identifying areas with poorer databases, this evidence map can also be used to inform future research efforts on topics that have been insufficiently studied. This evidence map does not (1) include evaluations of risk of bias or study sensitivity, (2) evaluate or report the results of studies regarding the existence or direction of potential associations between PCB exposure and health outcomes, (3) seek to describe every possible area of uncertainty that could benefit from further research, or (4) reach conclusions on causal relationships between PCB exposures and health effects. However, it does represent a potentially valuable resource for future researchers who wish to conduct additional analyses using a systematically curated database as a starting point.

## 2. Methods

### 2.1. Literature search

A literature search strategy was developed with library scientists, and PECO criteria were used to facilitate subsequent screening and categorization of literature into a systematic evidence map of PCB exposure and noncancer health endpoints (registered in 2019 via Zenodo, https://doi.org/10.5281/zenodo.3585771). Peer-reviewed literature was identified by searching PubMed (National Library of Medicine), Web of Science (Clarivate Analytics), and, prior to 2019, Toxline (National Library of Medicine). The literature search strategy relied on terms describing PCB mixtures and individual congeners (e.g., “polychlorinated biphenyls,” _“_Aroclor,” _“_PCB,” _“_tetrachlorobiphenyl”) to gather information on exposure to the chemicals of interest. Additional exposure terms were used to identify studies not indexed by the chemical name (e.g., “capacitor manufacturing workers,” “Yu-Cheng,” “New York State Angler Cohort”). These search terms were intentionally broad and did not prioritize studies in which exposure was quantified for individual participants; this was considered during screening of the literature. The detailed search strategies are presented in Tables S1A and a summary of search results is provided in Table S1B in Supplementary File 1. A list of all references retrieved through the literature searches is provided in Supplementary File 2, and search results by year are provided in Supplementary Files 3–9. The original search, conducted in 2015, was not restricted by publication date or language (Supplementary File 3). Literature search updates were conducted yearly through September 1, 2021 and were restricted to the 12-month period since the original search or the most recent update (Supplementary Files 4–9). Records identified through the original literature search were prioritized electronically as described below. Reference lists of health assessments published by federal, state, and international health agencies were searched to identify seed references (described below), but additional supplemental search strategies (e.g., citation mapping) were not applied. Twenty-five references were identified through recommendation by technical experts.

### 2.2. Electronic approaches used to refine a large database

**Fig. 1.**
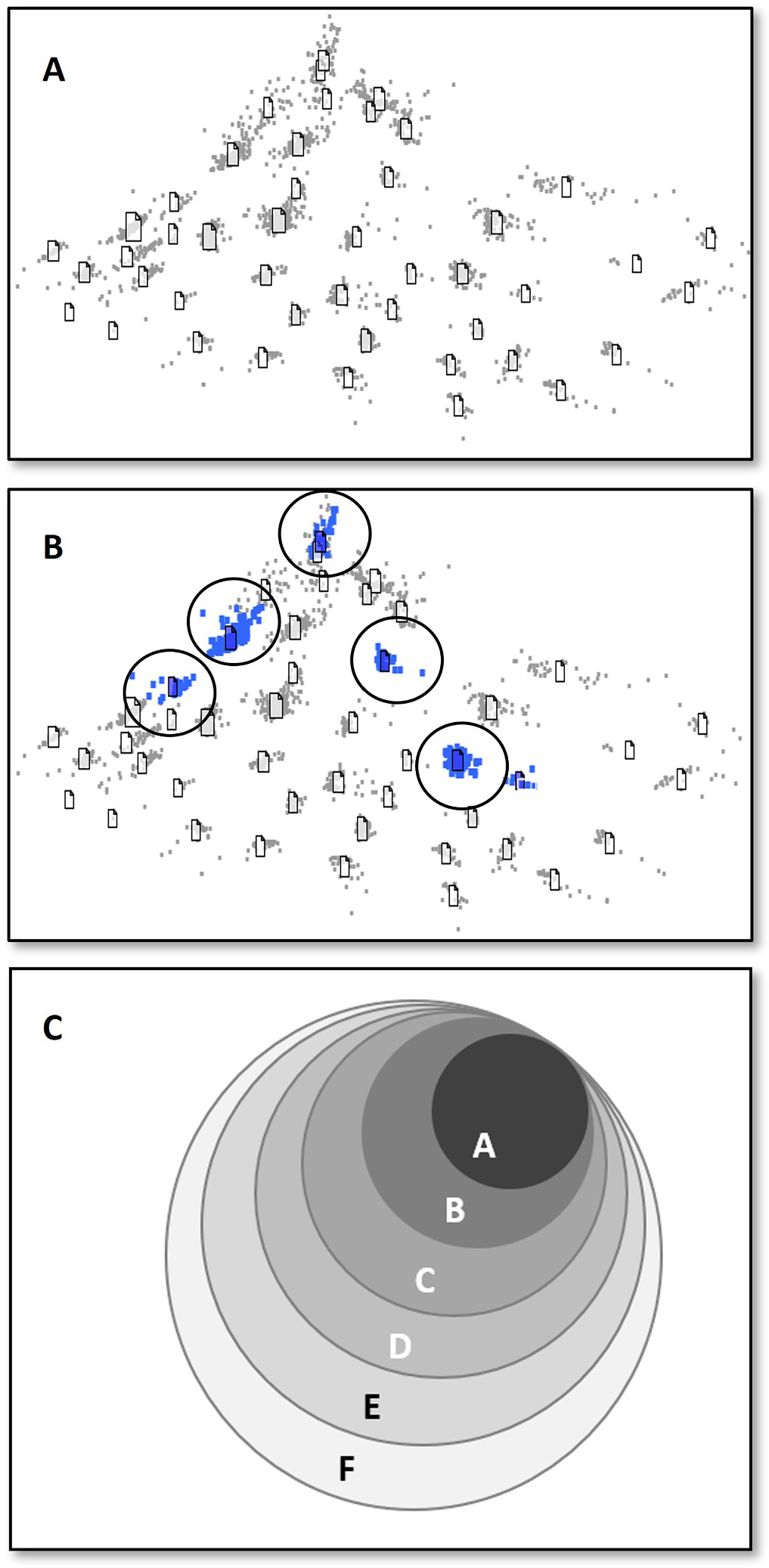
Illustration of electronic prioritization approaches. 1 A. Schematic illustration of electronic prioritization of literature depicting references clustered by similarity using natural language processing. 1 B. Illustration depicting clusters containing relevant seed references (circled blue clusters). Clusters were ranked by the number of seed studies included. 1C. Visualization of identified clusters. Clusters were organized into groups (A–F) on the basis of the number of approaches that identified the cluster such that Group A contains clusters harboring seed references identified by six approaches and Group F contains clusters harboring seed references identified by a single approach. All references in the top four groups (A–D) were manually screened for inclusion based on PECO criteria. Low scoring groups (E, F) were subjected to additional machine learning approaches to capture relevant references for manual screening. (For interpretation of the references to color in this figure legend, the reader is referred to the Web version of this article.)

Natural language processing (NLP) and machine learning (ML) techniques were employed to identify the most relevant literature for manual screening. Studies were prioritized using DoCTER, a Document Classification and Topic Extraction Resource (Varghese et al., 2018). Details of the NLP and ML methods are described elsewhere (Varghese et al., 2018, 2019). Briefly, 483 studies selected as having met the PECO criteria for inclusion in this review (Table 1) were designated as seed references (provided in Table S1C). Seed references are a set of relevant documents that are labeled and included in the larger collection of unclassified documents. Seed references function as tracers in the document prioritization process, helping identify documents with similar content. For this review, seed references were identified during problem formulation (US EPA, 2015), which was largely on the basis of a survey of references cited in health assessments published by federal, state, and international health agencies, most notably ATSDR (2000). Seed studies were included in the corpus of references identified by keyword searches and used to prioritize studies using an ML method called supervised clustering. In Phase 1 of a two-phased prioritization approach, titles and abstracts of all references in the corpus were represented as a mathematical matrix using an NLP transformation and then organized into clusters on the basis of semantic similarity using clustering algorithms as depicted in Fig. 1A. Two clustering algorithms (k-means, nonnegative matrix factorization) were applied using cluster sizes of 10, 20, or 30 references for a total of 6 different clustering approaches. Clusters harboring seed references were identified as illustrated in Fig. 1B.

In each approach, clusters were ranked in decreasing order of the number of seed studies in each cluster, and clusters were accepted in order until 90% of the total set of seed studies was captured. A 90% threshold was selected because it provided an optimal balance between the statistical measures of recall (fraction of relevant studies retrieved) and precision (fraction of relevant studies among the retrieved studies) (Varghese et al., 2018). Higher thresholds would have resulted in diminished precision and a considerably higher manual screening burden given the size of the original literature database. This method was repeated for all six clustering approaches; thus, a given study could have appeared in one of the accepted clusters (and thus appear with the greatest fraction of the seed studies) in anywhere from zero to six of the approaches. Clusters containing seed references were grouped by the number of approaches in which they were identified (Groups A–F, Fig. 1C). Studies that appeared in groups A–D (representing 6, 5, 4, or 3 approaches, Fig. 1C) were subjected to manual title and abstract-level screening, as described below. Screened studies from Phase 1 were used to train the ML model in Phase 2.

In Phase 2, a supervised ML algorithm (support vector machines (Varghese et al., 2018)) was used to predict relevance for those studies in the remaining groups of clusters that appeared in one or two approaches (groups E and F, Fig. 1C). Also included in this approach was one group of studies excluded from initial clustering until abstracts were recovered and a second group of studies with titles only. The training data set for this secondary analysis is distinct from the seed references mentioned above and included all studies manually screened in Phase 1; the training data set for supervised ML thus included examples of studies that met PECO and those that did not. Studies predicted to be relevant in Phase 2 by the supervised ML algorithm were subjected to manual title and abstract-level screening. Details of the DoCTER prioritization strategy are presented in Tables S1D and a list of studies prioritized using DoCTER supervised clustering and ML approaches is provided in Table S1E.

**Fig. 2.**
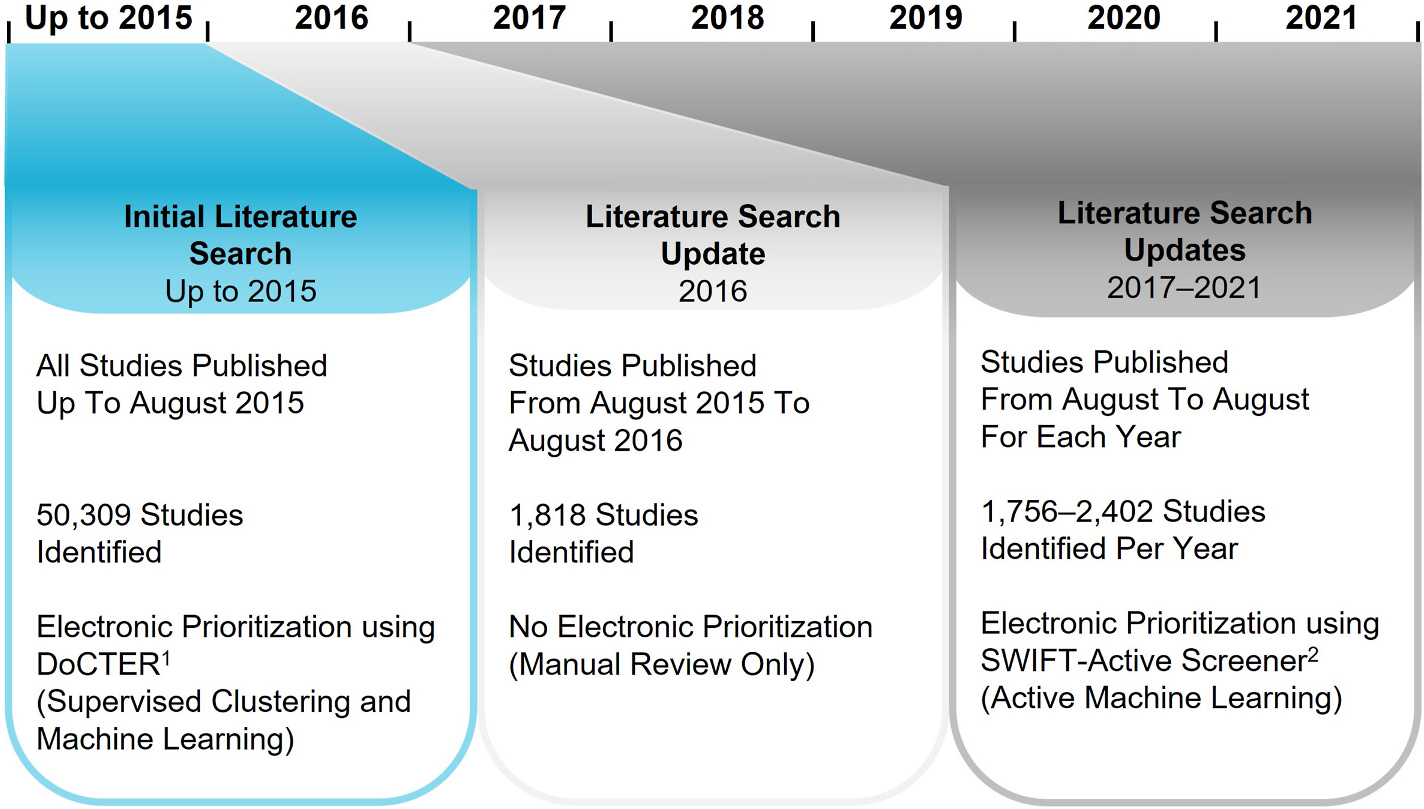
Chronology of prioritization approaches applied to PCB literature search results. The initial 2015 literature search used DoCTER, an electronic prioritization approach described in Section 2.2 and in ICF (2019). The literature updates from 2017 to 2021 utilized SWIFT-Active Screener, another electronic prioritization program and fully described in Sciome (2021).

Because fewer references were identified in yearly search updates, alternative approaches were used to prioritize the literature for screening (summarized in Fig. 2). References identified in 2016 were directly subjected to manual screening without electronic prioritization. References identified after August 2016 were prioritized using SWIFT-Active Screener (Sciome, 2021), a web-based software application integrated with electronic prioritization using ML and statistical approaches. For each literature update, manually reviewed studies were used to train the model, which is updated iteratively, thus reducing manual screening efforts (Howard et al., 2020).

### 2.3. Literature screening

#### 2.3.1. Title and abstract screening of the literature

Prioritized records were combined with smaller groups of records, including seed references, records identified through literature search updates, and references suggested by technical experts, into a single database (Table S1E and S1F). The literature was then manually screened in two steps to determine whether individual studies should be included or excluded as a primary source of health endpoint data on the basis of PECO criteria shown in Table 1. Step 1 consisted of title and abstract screening, while Step 2 involved full-text screening.

In Step 1, two trained screeners independently conducted a manual title and abstract review using structured forms developed in DRAGON (a modular database with integrated literature evaluation and screening tools developed for systematic review) (ICF, 2018) to identify records that appeared to meet the PECO criteria. For citations with no abstract, articles were screened based on title relevance. Screening conflicts were resolved by a third reviewer. Each study was categorized to one of the following bins: Relevant to Hazard Identification in Humans, Relevant to Hazard Identification in Animals (nonhuman mammals only), Potentially Relevant Supplemental Material, or Not Relevant. Potentially relevant supplemental material included toxicokinetic studies, studies describing pharmacokinetic models for PCB congeners and mixtures, and mechanistic studies. Additional records tracked as potentially relevant supplemental material included conference abstracts, secondary data sources (e.g., reviews, agency assessments), non-English-language studies, exposure studies unrelated to health endpoints, and human case reports or case series. The tags used for study categorization are summarized in Tables S1G, S1H, and S1I. Categories of potentially relevant supplemental material are shown in Table S1I.

References retrieved through August 2016 were screened and tagged using DRAGON. Screening decisions and study metadata recorded in DRAGON (v. 03-25-2016) were recently moved to a second generation, web-based platform rebranded as litstream^™^ (ICF, 2019). References identified in search updates after August 2016 were screened in SWIFT-Active Screener until the software indicated a likelihood of 95% that all relevant studies had been captured. This threshold is comparable to human error rates (Bannach-Brown et al., 2018; Howard et al., 2016; Cohen et al., 2006) and is used as a metric to evaluate ML performance. A summary of literature prioritized using SWIFT-Active Screener is provided in Table S1F.

To validate the application of clustering and ML algorithms, a subset of nonprioritized studies was also randomly selected for manual title and abstract-level review.

#### 2.3.2. Full-text screening of the literature

Records not excluded or considered potentially relevant supplemental material on the basis of the title and abstract advanced to full-text review using litstream. Full-text copies of these potentially relevant records were retrieved and independently assessed by two screeners to confirm eligibility according to the PECO criteria. Screening conflicts were resolved by a third reviewer. Seed references, which had been identified as meeting PECO during problem formulation (US EPA, 2015) were also included in the full-text screen to categorize these records.

In addition to confirming that studies met PECO criteria, the health endpoints investigated in each study were identified using structured forms in litstream. Health endpoints were organized by organ/system as outlined in (Thayer et al., 2022): Cardiovascular, Dermal, Developmental, Endocrine, Gastrointestinal, Hematopoietic, Hepatobiliary, Immune System, Metabolic, Musculoskeletal, Nervous System, Ocular, Reproductive, Respiratory, and Urinary System. These categories were chosen because of their potential to include specific noncancer health endpoints that could be affected by PCB exposure at levels relevant to those experienced in the general population. Assignment of specific endpoints into each of the organs/systems was made by one or more coauthors based on their primary areas of expertise. Some studies evaluated multiple endpoints and so were assigned to multiple organs/systems. We recognize that there is crosstalk among many physiological systems, which can complicate the categorization of endpoints; specific information on which endpoints are included in each organ/system is provided in Section 3.2.2. Studies focused entirely on cancer or frank toxicity, including mortality of unknown cause and wasting, although not the focus of this review, were considered potentially relevant supplemental material. Furthermore, at this stage, because the focus of the review is on potential effects of exposure to complex PCB mixtures similar to those found in the environment, toxicological studies in which mammals were exposed only to individual PCB congeners or to mixtures comprising fewer than four congeners were considered potentially relevant supplemental material.

On the basis of the results for the full-text review, summary-level, sortable lists of relevant literature were created for human and animal (nonhuman mammalian) studies for each organ/system. Fundamental study design information (e.g., study population, exposure assessment/design, health endpoints evaluated) was extracted for each study in Microsoft Excel by one individual and independently reviewed by at least one additional individual.

## 3. Results and discussion

### 3.1. Literature search and screen

#### 3.1.1. Literature searches (2015–2021)

**Fig. 3.**
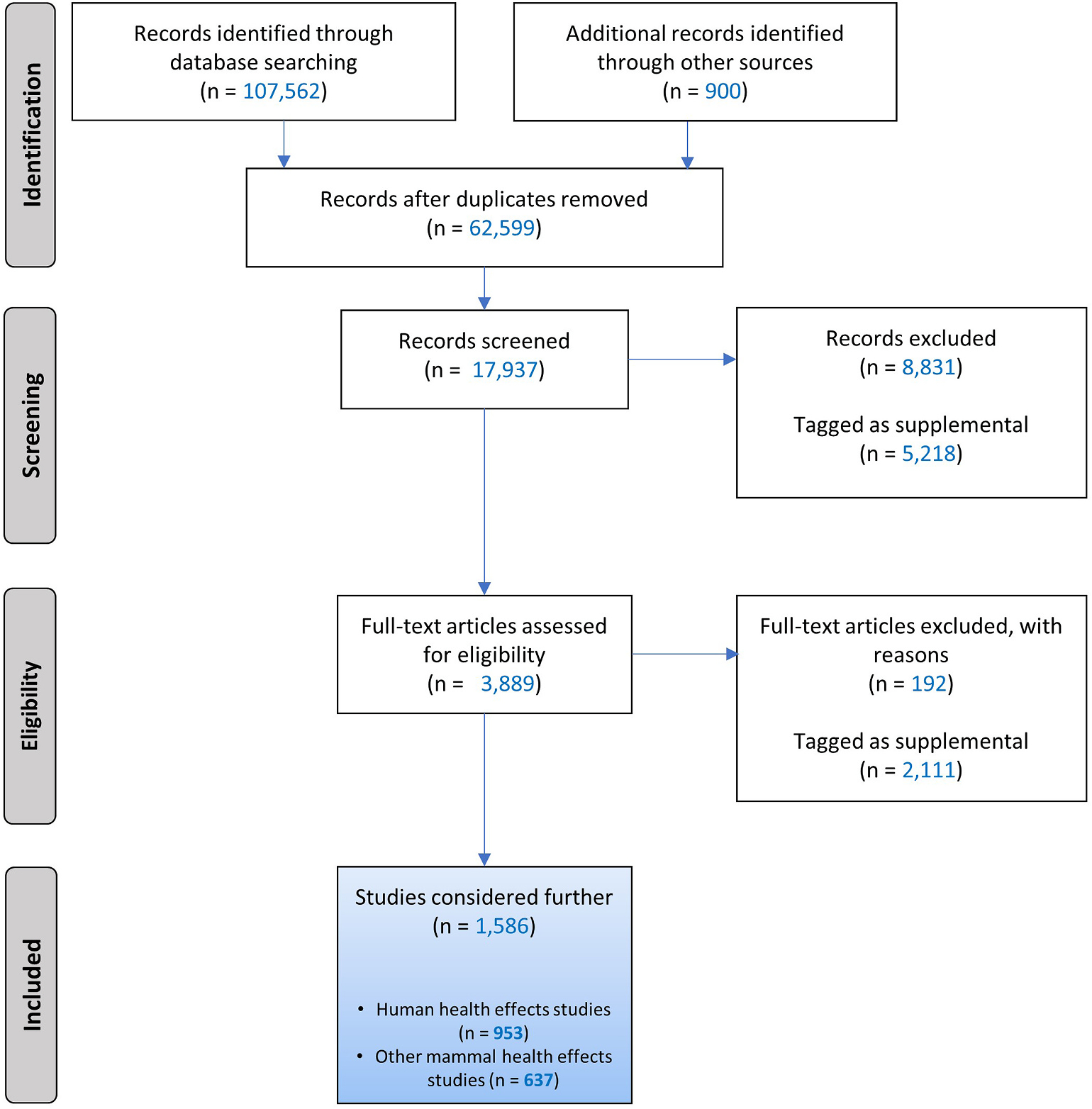
Literature search flow diagram for PCBs. Searches were conducted up to August 31, 2021, in PubMed, Web of Science, and, prior to 2019, also Toxline. References were identified by technical experts or through supplemental searches, including seed references (records identified through other sources). During screening, records were prioritized using machine learning tools (DoCTER for references identified in 2015–2016 database searches and SWIFT-Active Screener for 2017–2021). Excluded records are those studies not meeting PECO criteria. Records tagged as supplemental includes potentially relevant supplemental material such as conference abstracts, reviews, and non-English studies that met PECO and studies on PECO-related topics (e.g., toxicokinetic or mechanistic studies). Some studies examined health endpoints in both humans and other mammals.

The results of the literature searches and screens for literature published through August 31, 2021, are summarized in the Literature Flow Diagram presented in Fig. 3. After duplicate removal, 50,309 studies were identified from the initial literature search conducted in 2015. Yearly literature search updates were conducted from 2016 to 2021, yielding between 1756 and 2402 unique studies per year, which added 11,390 studies. Thus, 61,699 studies were identified through database searching after duplicate removal.

#### 3.1.2. Electronic prioritization for manual review

Manual screening of the entire database was time and cost prohibitive because of the vast number of studies. Therefore, electronic prioritization approaches were implemented to identify studies most likely to meet the PECO criteria (Table 1). Results and prioritization strategies are summarized in Fig. 2 and Table S1B.

As described above for the initial literature search conducted in 2015, both supervised clustering and ML approaches were used to prioritize studies for manual screening. The number of studies identified using electronic prioritization methods is summarized in Table 2.

Studies not manually reviewed included those not identified by any of the clustering approaches or those identified by one or two approaches and predicted not to be relevant during the ML phase. Collectively, clustering and ML approaches using DoCTER identified 11,177 studies from the initial literature search conducted in 2015 as high priority studies for manual review. Review of a randomly selected subset of nonprioritized studies demonstrated that less than 10% of nonprioritized studies were relevant based on PECO criteria, indicating that these approaches captured at least 90% of the literature relevant to informing associations between PCB exposure and health endpoints. In 2016, all 1818 unique studies were manually reviewed. Of the 9572 studies retrieved in 2017–2021, 4042 studies were prioritized using SWIFT-Active Screener and were manually screened. Thus, 17,037 (27.6%) of the 61,699 studies retrieved through literature searches were manually reviewed.

#### 3.1.3. Literature screening

In addition to electronically prioritized studies, 900 other studies were manually reviewed. These included 483 seed references and 417 studies identified by technical experts or through supplemental literature searches conducted to identify information on PCB toxicokinetics or modes of action. The manual review thus included 17,937 studies. Of these, 3889 were identified as potentially relevant and subjected to full-text review and categorization by health endpoint. After full-text review and categorization, 1586 studies were included in literature summary tables organized by organ/system, with one summary table per system for human studies and a second for animal studies (nonhuman mammals only). A total of 953 human studies and 637 nonhuman mammalian studies were evaluated for health endpoints and exposure to PCBs.

Most studies evaluated more than one type of health endpoint, so the numbers of studies for each system are not expected to sum to the total number of studies, nor are the numbers of studies of each health endpoint expected to sum to the total number of studies for the relevant organ/system. Furthermore, sometimes results from a single research project are reported in multiple publications. Different publications might focus on different subsets of data, use different statistical or other analytical approaches, or update results published from earlier stages of data collection. For example, a study of women with and without endometriosis first described by Buck Louis et al. (2005) was later reanalyzed using different statistical techniques (Roy et al., 2012; Gennings et al., 2010). When the same results were reported in multiple publications or when some publications reported results on the basis of less complete data, we considered those data only as reported in the most up-to-date or most complete publication. Even so, this multiplicity of published reports is important to note when interpreting numbers of studies identified by the literature search and screening process and evaluation of the adequacy of the database to support hazard conclusions. To help readers navigate through the literature and understand the basis for our study counts, we have developed interactive visualizations that allow for identification of the individual references included for each organ/system (https://hawcprd.epa.gov/summary/assessment/100500282/visuals/; underlying data provided in Table S1J). These visualizations can also be used to filter references by study design characteristics of interest to generate customized reference lists and counts of studies that we do not include in this report. This paper provides some preliminary analysis of the strengths and weaknesses of the existing databases for various health endpoints evaluated in relation to PCB exposure. Our focus is on the utility of such information for future PCB hazard assessments. However, we hope that researchers will query these visualizations and the underpinning database to inform a much wider variety of research questions related to PCB exposures and health effects.

### 3.2. Summary of database of human and nonhuman mammalian studies

**Fig. 4.**
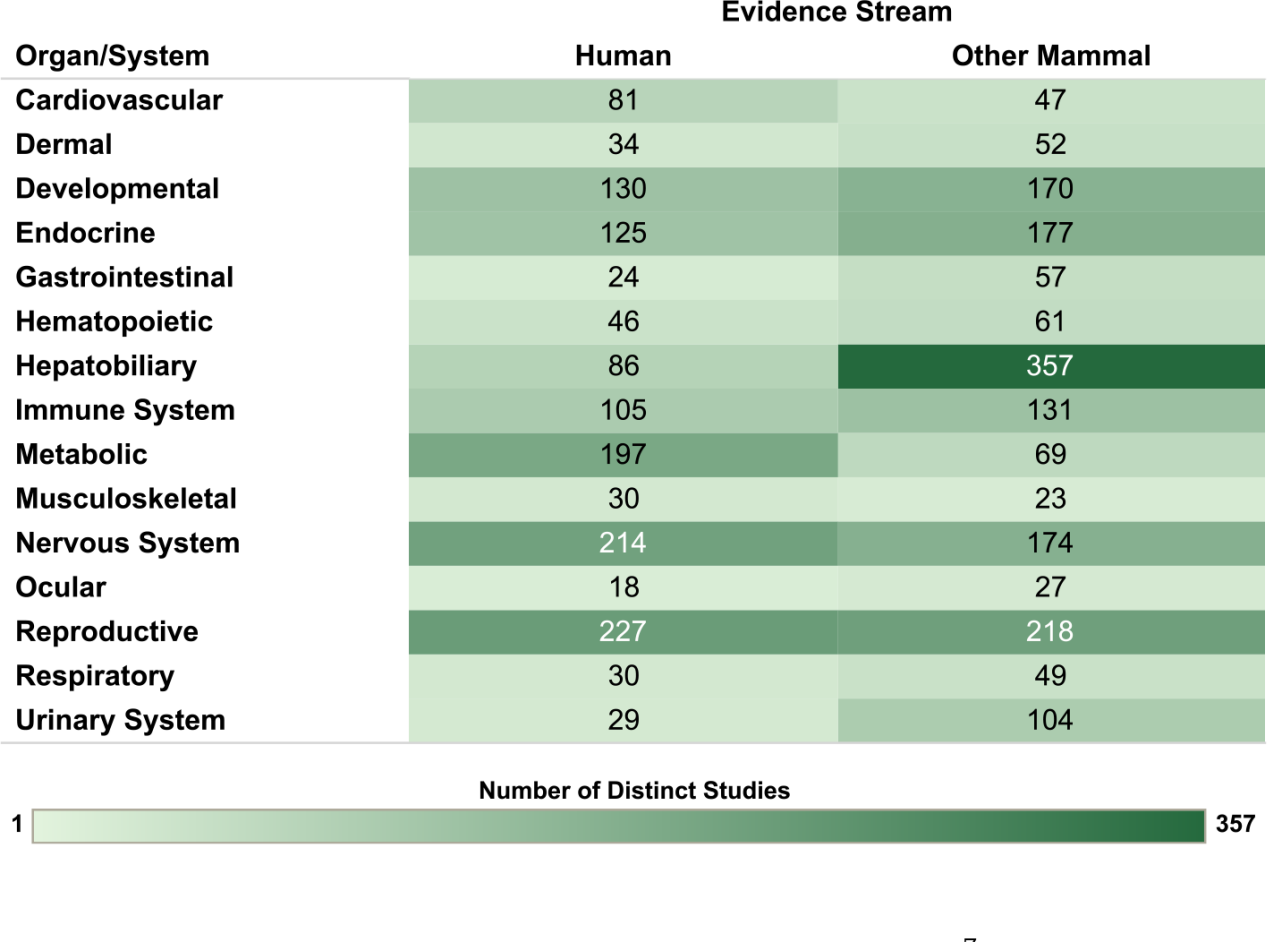
Overview of Human and Other Mammalian Studies Across Organs/Systems. Summary of the database of studies evaluating exposures to PCB mixtures and health endpoints organized by system. Lists of studies included in each count can be accessed via the online interactive version of this figure (https://hawc.epa.gov/summary/visual/assessment/100500282/OverviewAllStudies/). The online figure can be expanded to include information by endpoint category. Shading intensity corresponds with the number of studies in each category, from 1 to 357, which is the maximum number of studies in any category.

Overview figures were generated from the interactive visualizations described above (https://hawcprd.epa.gov/summary/assessment/100500282/visuals/) to summarize the database of human and nonhuman mammalian studies that met PECO criteria. Fig. 4 presents an overview of studies across the 15 organs/systems.

**Fig. 5.**
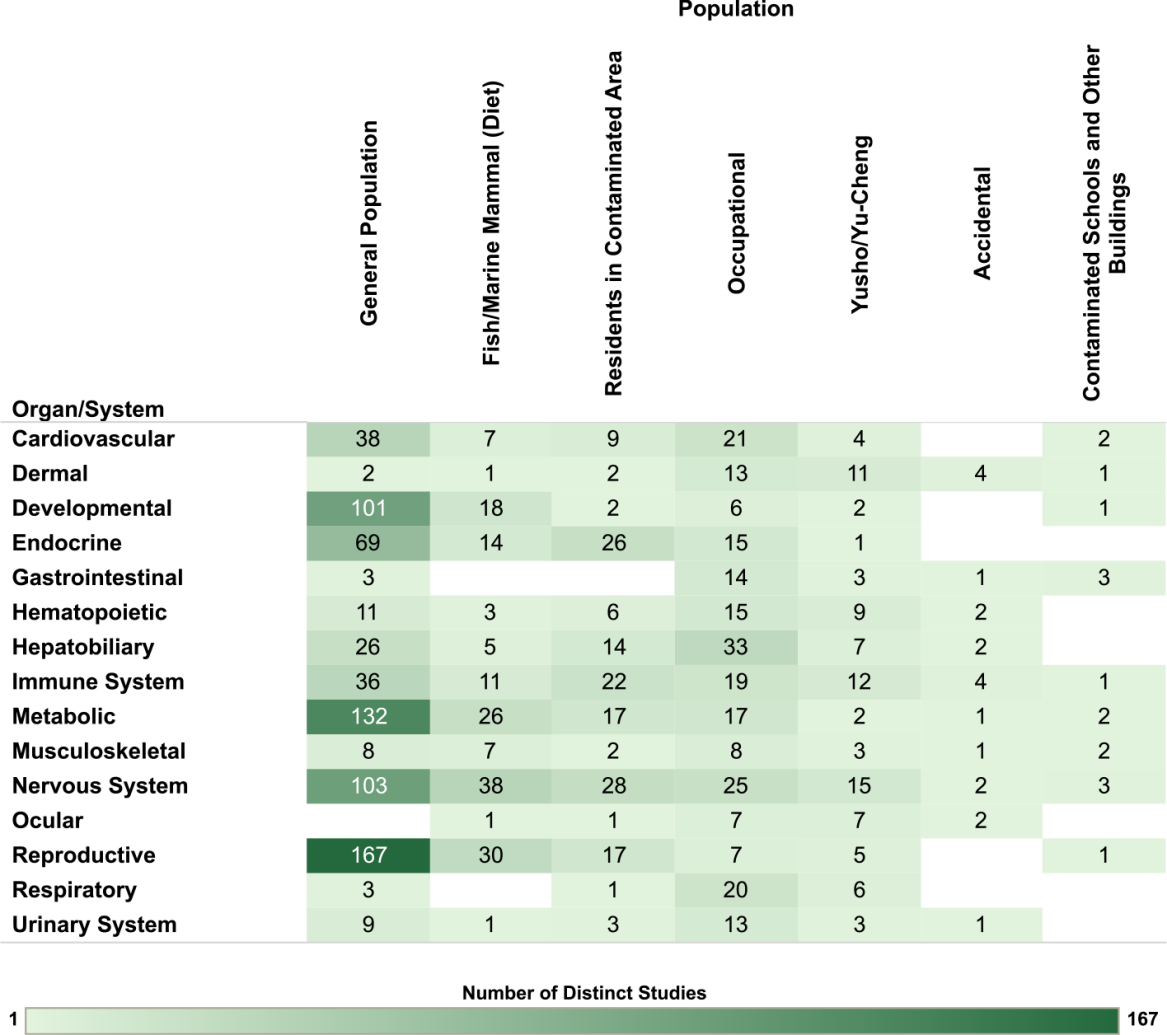
Overview of Human Studies by Organ/System and Population. Summary of the database of human studies evaluating exposures to PCB mixtures and health endpoints organized by system and population. Lists of studies included in each count can be accessed via the online interactive version of this figure (https://hawc.epa.gov/summary/visual/assessment/100500282/OverviewHumanStudies/). The online figure can be expanded to include information by endpoint category and can be filtered by organ/system (options: cardiovascular, dermal, developmental, endocrine, gastrointestinal, hematopoietic, hepatobiliary, immune system, metabolic, musculoskeletal, nervous system, ocular, reproductive, respiratory, urinary system), study design (options: case-control, cohort, cross-sectional, other), population (options: accidental, contaminated schools and other buildings, fish/marine mammal (diet), general population, occupational, residents in contaminated area, Yusho/Yu-Cheng), sex (relevant only for reproductive endpoints; options: couple, female, male), and exposure metric (options: adipose tissue, blood, breast milk, child blood, cord blood, dietary estimates, fish consumption, maternal blood, occupational/JEM, other metric [includes dust and modeled estimates], other tissue). Shading intensity corresponds with the number of studies in each category, from 1 to 167, which is the maximum number of studies in any category. JEM = job exposure matrix.

**Fig. 6.**
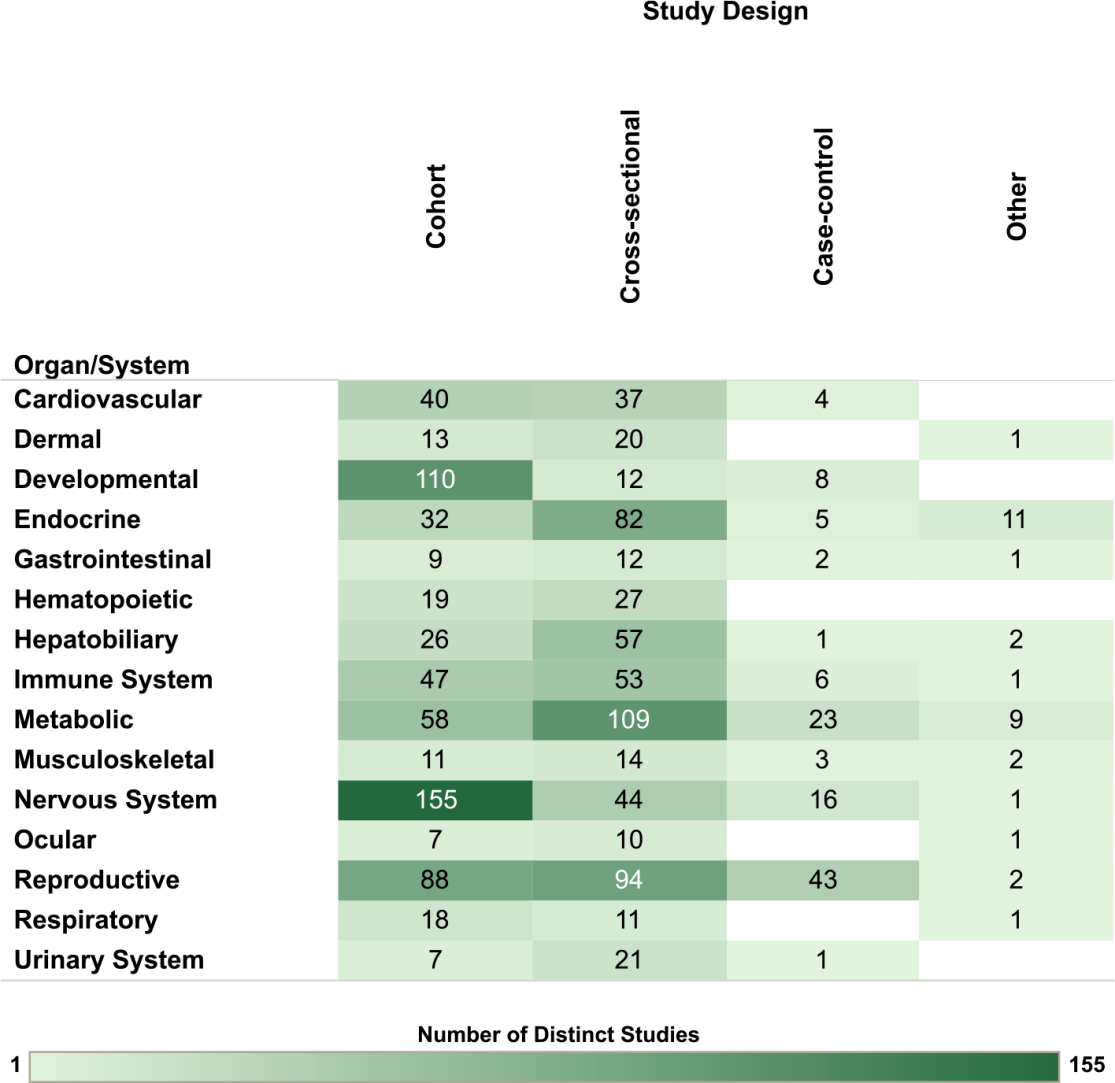
Overview of Human Studies by Organ/System and Study Design. Summary of the database of human studies evaluating exposures to PCB mixtures and health endpoints organized by system and study design. Lists of studies included in each count can be accessed via the online interactive version of this figure (https://hawc.epa.gov/summary/visual/assessment/100500282/OverviewHumanStudies/). The online figure can be expanded to include information by endpoint category and can be filtered by organ/system (options: cardiovascular, dermal, developmental, endocrine, gastrointestinal, hematopoietic, hepatobiliary, immune system, metabolic, musculoskeletal, nervous system, ocular, reproductive, respiratory, urinary system), study design (options: case-control, cohort, cross-sectional, other), population (options: accidental, contaminated schools and other buildings, fish/marine mammal (diet), general population, occupational, residents in contaminated area, Yusho/Yu-Cheng), sex (relevant only for reproductive endpoints; options: couple, female, male), and exposure metric (options: adipose tissue, blood, breast milk, child blood, cord blood, dietary estimates, fish consumption, maternal blood, occupational/JEM, other metric [includes dust and modeled estimates], other tissue). Shading intensity corresponds with the number of studies in each category, from 1 to 155, which is the maximum number of studies in any category. JEM = job exposure matrix.

**Fig. 7.**
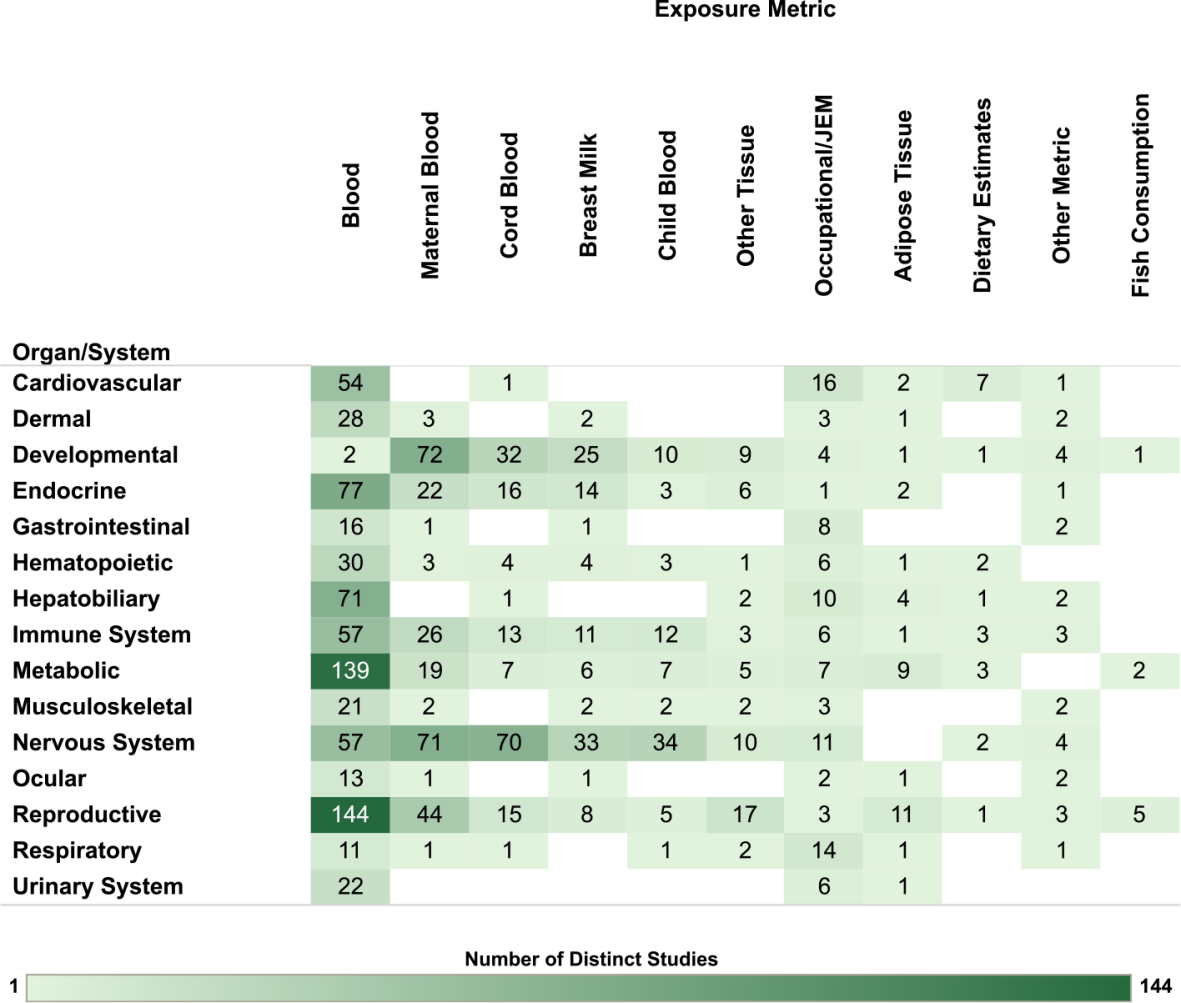
Overview of Human Studies by Organ/System and Exposure Metric. Summary of the database of human studies evaluating exposures to PCB mixtures and health endpoints organized by system and exposure metric. Lists of studies included in each count can be accessed via the online interactive version of this figure (https://hawc.epa.gov/summary/visual/assessment/100500282/OverviewHumanStudies/). The online figure can be expanded to include information by endpoint category and can be filtered by organ/system (options: cardiovascular, dermal, developmental, endocrine, gastrointestinal, hematopoietic, hepatobiliary, immune system, metabolic, musculoskeletal, nervous system, ocular, reproductive, respiratory, urinary system), study design (options: case-control, cohort, cross-sectional, other), population (options: accidental, contaminated schools and other buildings, fish/marine mammal (diet), general population, occupational, residents in contaminated area, Yusho/Yu-Cheng), sex (relevant only for reproductive endpoints; options: couple, female, male), and exposure metric (options: adipose tissue, blood, breast milk, child blood, cord blood, dietary estimates, fish consumption, maternal blood, occupational/JEM, other metric [includes dust and modeled estimates], other tissue). Shading intensity corresponds with the number of studies in each category, from 1 to 144, which is the maximum number of studies in any category. JEM = job exposure matrix.

For the human studies, the database is organized by organ/system and study population in Fig. 5, by organ/system and study design in Fig. 6, and by organ/system and PCB exposure metric in Fig. 7. When describing exposure metrics, the term “blood” was used to broadly capture measurements made in whole blood or any fraction (serum or plasma). Multiple blood metrics are identified to indicate the lifestage represented by the measurement (i.e., blood (collected from adults outside the context of pregnancy or lactation), child blood, maternal blood, cord blood). These figures are also linked to interactive graphics, which allow users to filter by specific details (organ/system, study design, population, exposure matrix).

**Fig. 8.**
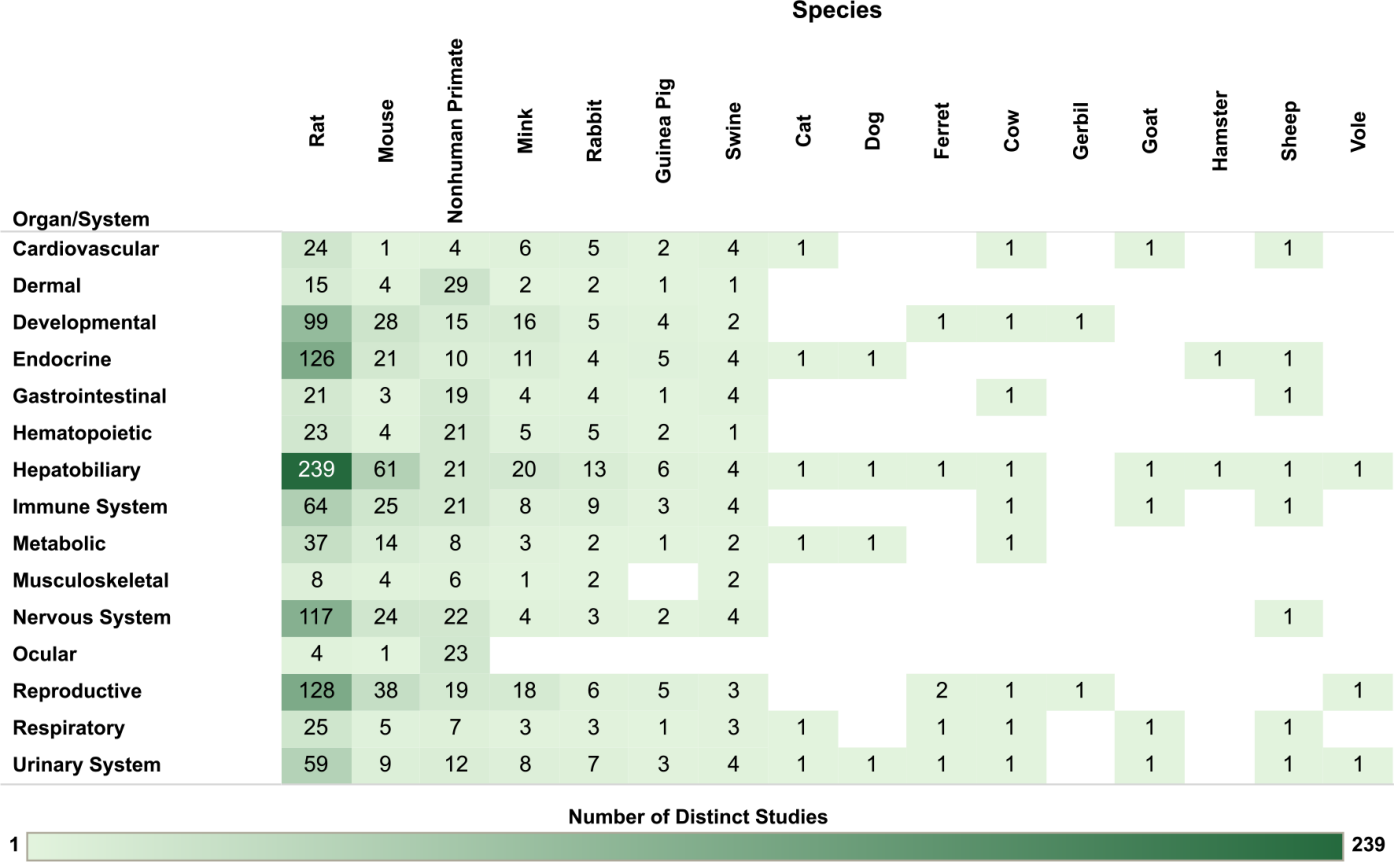
Overview of Nonhuman Mammalian Studies by Organ/System and Species. Summary of the database of studies in nonhuman mammals evaluating exposures to PCB mixtures and health endpoints organized by system and species. Lists of studies included in each count can be accessed via the online interactive version of this figure (https://hawc.epa.gov/summary/visual/assessment/100500282/OverviewNonhumanMammalStudies/). The online figure can be expanded to include information by endpoint category and can be filtered by organ/system (options: cardiovascular, dermal, developmental, endocrine, gastrointestinal, hematopoietic, hepatobiliary, immune system, metabolic, musculoskeletal, nervous system, ocular, reproductive, respiratory, urinary system), exposure duration/life stage (options: acute [single dose], chronic, developmental, NR, short-term, subchronic), species (options: cat, cow, dog, ferret, gerbil, goat, guinea pig, hamster, mink, mouse, nonhuman primate, rabbit, rat, sheep, swine, vole), sex (relevant only for reproductive endpoints; options: female, male, pair), and exposure route (options: dermal, inhalation, injection, oral). Shading intensity corresponds with the number of studies in each category, from 1 to 239, which is the maximum number of studies in any category. NR = not reported.

**Fig. 9.**
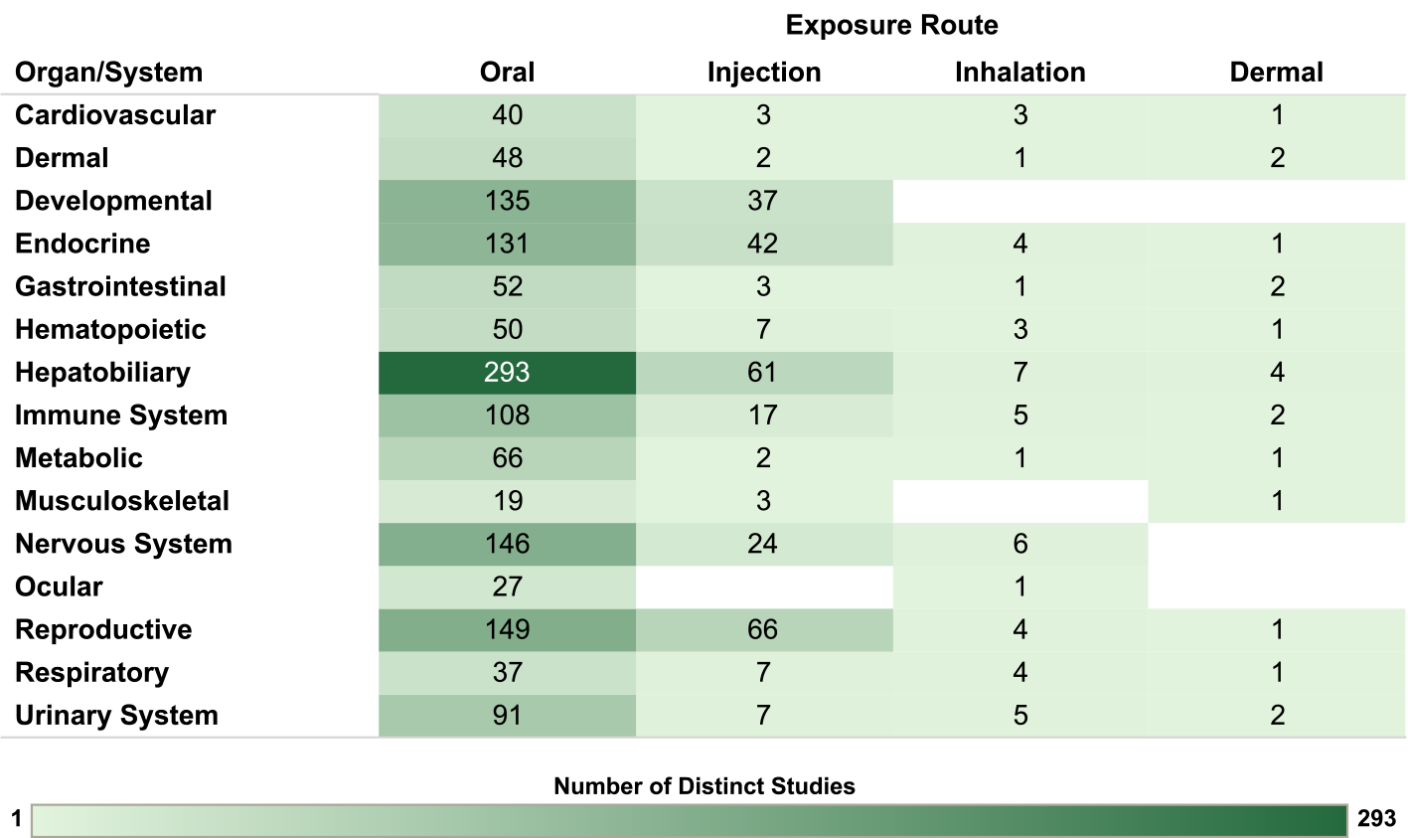
Overview of Nonhuman Mammalian Studies by Organ/System and Exposure Route. Summary of the database of studies in nonhuman mammals evaluating exposures to PCB mixtures and health endpoints organized by system and exposure route. Lists of studies included in each count can be accessed via the online interactive version of this figure (https://hawc.epa.gov/summary/visual/assessment/100500282/OverviewNonhumanMammalStudies/).The online figure can be expanded to include information by endpoint category and can be filtered by organ/system (options: cardiovascular, dermal, developmental, endocrine, gastrointestinal, hematopoietic, hepatobiliary, immune system, metabolic, musculoskeletal, nervous system, ocular, reproductive, respiratory, urinary system), exposure duration/life stage (options: acute [single dose], chronic, developmental, NR, short-term, subchronic), species (options: cat, cow, dog, ferret, gerbil, goat, guinea pig, hamster, mink, mouse, nonhuman primate, rabbit, rat, sheep, swine, vole), sex (relevant only for reproductive endpoints; options: female, male, pair), and exposure route (options: dermal, inhalation, injection, oral). Shading intensity corresponds with the number of studies in each category, from 1 to 293, which is the maximum number of studies in any category. NR = not reported.

**Fig. 10.**
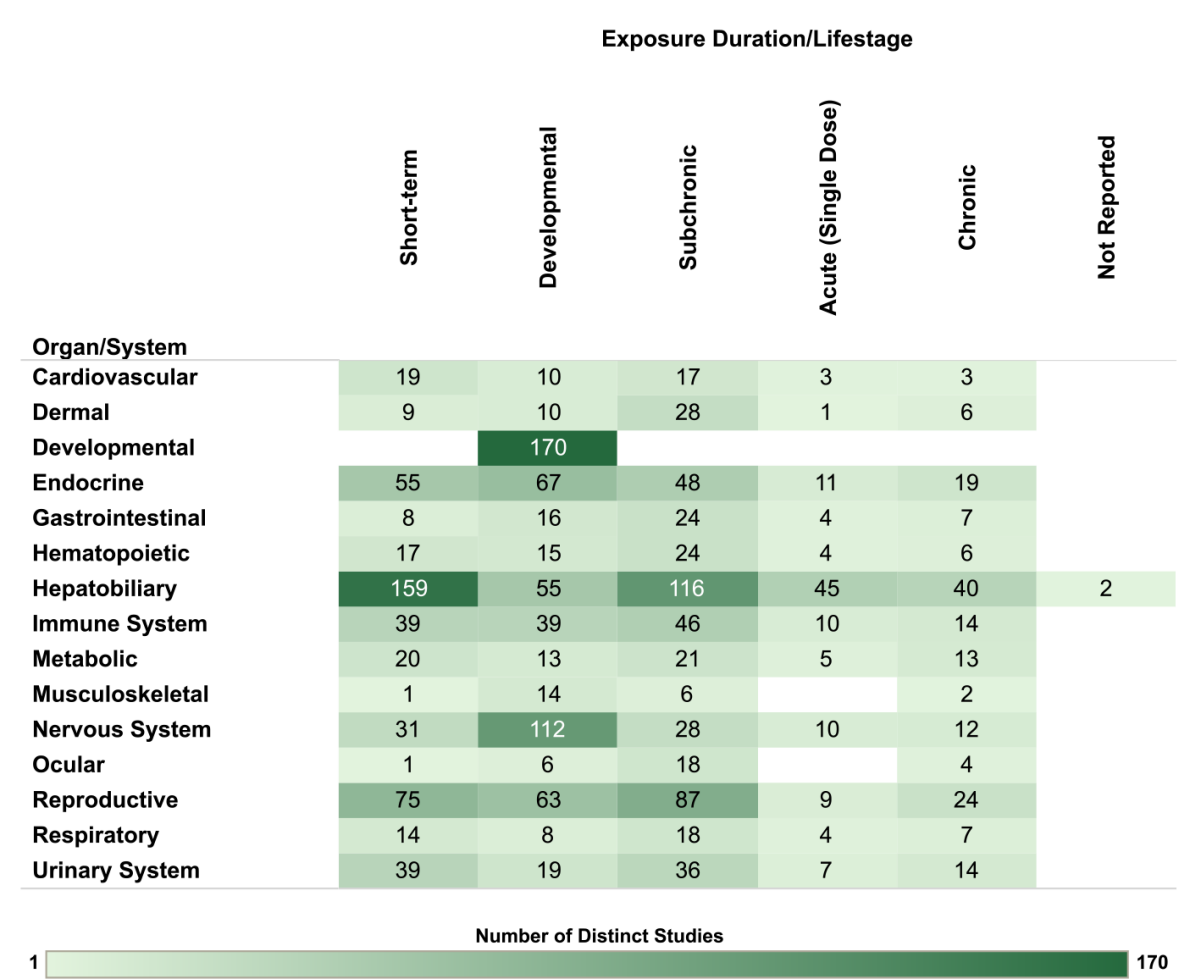
Overview of Nonhuman Mammalian Studies by Organ/System and Exposure Duration/Lifestage. Summary of the database of studies in nonhuman mammals evaluating exposures to PCB mixtures and health endpoints organized by system and exposure duration/lifestage. Lists of studies included in each count can be accessed via the online interactive version of this figure (https://hawc.epa.gov/summary/visual/assessment/100500282/OverviewNonh umanMammalStudies/). The online figure can be expanded to include information by endpoint category and can be filtered by organ/system (options: cardiovascular, dermal, developmental, endocrine, gastrointestinal, hematopoietic, hepatobiliary, immune system, metabolic, musculoskeletal, nervous system, ocular, reproductive, respiratory, urinary system), exposure duration/life stage (options: acute [single dose], chronic, developmental, NR, short-term, subchronic), species (options: cat, cow, dog, ferret, gerbil, goat, guinea pig, hamster, mink, mouse, nonhuman primate, rabbit, rat, sheep, swine, vole), sex (relevant only for reproductive endpoints; options: female, male, pair), and exposure route (options: dermal, inhalation, injection, oral). Shading intensity corresponds with the number of studies in each category, from 1 to 170, which is the maximum number of studies in any category. NR = not reported.

For the nonhuman mammalian studies, the database is organized by organ/system and animal species in Fig. 8, by organ/system and exposure route in Fig. 9 and by organ/system and exposure duration/lifestage in Fig. 10. The interactive graphics corresponding with these figures can be filtered and expanded based on organ/system, endpoint category, exposure duration/lifestage, species, and exposure route. For reproductive endpoints, users can also filter both the human and nonhuman mammalian interactive graphics by sex.

#### 3.2.1. General considerations

One potential use for this evidence map is to provide a starting point for future health hazard assessments of PCB mixtures. Such assessments could include full systematic reviews of the evidence for PCB effects on specific types of health outcomes. The amount of time, resources, and level of effort required to develop a full systematic review are substantial. For many of the organs/systems in this review, the database is extensive and diverse; this presents challenges for future analyses and for expeditious development of assessments to inform efforts to protect human health. A practical preliminary step for prioritizing organs/systems for systematic review is to identify health endpoints with databases that are sufficiently large and informative to potentially support meaningful conclusions about causal relationships with PCB exposure. This evidence map is designed to facilitate the identification of such databases that could be further evaluated through subsequent systematic reviews, which would include study evaluation and evidence synthesis and integration (US EPA, 2019). Determination of causal relationships between exposures and health effects forms the basis for hazard identification, which is a fundamental step in human health risk assessment (NRC, 1983).

In the PCBs database, the studies were primarily of the general population, which is expected to be exposed to relatively low levels of PCBs, predominantly through dietary exposure (e.g., ingestion of fatty foods, such as fish, meat, and dairy) with some contribution from inhalation and dermal exposure (Weitekamp et al., 2021). There were some studies in populations where exposures were greatly increased, or where the primary exposure routes and sources were different from the general population. These include populations with greater contribution from inhalation and dermal exposure routes through residence near a contaminated site, time spent in contaminated residences and buildings, or occupational exposures, populations with greater magnitude of dietary exposure from fish/marine mammal diets or Yusho/Yu-Cheng exposures, or other greatly increased (accidental) PCB exposure. In the Yusho (Japan, 1968) and Yu-Cheng (Taiwan, 1979) incidents, people were accidently exposed to PCBs and their degradation products, polychlorinated dibenzofurans (PCDFs), via contaminated rice bran oil. As described by ATSDR (2000) and Kunita et al. (1985), PCDFs and dioxin-like PCBs could affect the same health outcomes because they share a common mode of action mediated by the AhR. Such co-occurring exposures (either harmful [e.g., methylmercury] or beneficial [e.g., long-chain fatty acids] (Paliwoda et al., 2016; Turyk et al., 2012) can complicate evaluation of health endpoints if they are highly correlated with PCBs and the endpoint of interest in the study population.

The most common types of study design for human studies of PCB exposure were cohort and cross sectional followed by case-control and other study designs. We note that while we designated study design using these broad categories for the purpose of this review, study design does not always fall cleanly into these bins. For example, as described above, many studies evaluate health endpoints following unintended exposure to PCBs and PCDFs in the Yusho and Yu-Cheng populations. Evaluation of these studies is complicated by the fact that membership in respective health registries is often used to confer “exposed” status, while measurements in biological tissues were taken at various time points after the exposure occurred, sometimes concurrent with the endpoint measure and sometimes before or afterward. For this review, studies of Yusho and Yu-Cheng are generally considered to be cohort studies, but specific cases are noted when exposure measures are interpreted differently on the basis of timing relative to outcome ascertainment.

Most human studies in the database characterized PCB exposure using measures of PCB congeners or their metabolites in biological matrices. Measurements were most frequently made in plasma/serum collected from adults outside the context of pregnancy or lactation or, for studies of mother-infant pairs, in maternal plasma/serum, umbilical cord plasma/serum, human milk, or child blood. PCB measurements in adipose and other tissues are much less common. Some studies assessed PCB exposure using dietary estimates or fish consumption, occupational/job exposure matrices, or other metrics. There are some limitations associated with human exposure assessments based on PCB measurements in biological samples (Christensen et al., 2021). For example, because many PCBs are lipophilic, measurement of PCB levels in body tissues and resulting estimates of associations with health endpoints can be influenced not only by differences in body composition between individuals or in the same individuals over time, but also by temporal trends (overall and by age group) and population-level changes in body composition over time (Wood et al., 2016). Despite such limitations, with careful interpretation, studies conducted using PCB measurements in blood or other matrices provide valuable data to inform assessments of human health hazard, especially in combination with information from controlled exposure studies conducted in laboratory animals.

Many studies evaluate mortality rates, or cause-specific mortality rates, as a health endpoint. This is particularly common in the PCB literature for occupationally exposed populations. These estimates can provide valuable information when the cause-of-death coding is likely to be sensitive and specific, and when an appropriate comparator population is selected. In many cases, however, cause-of-death coding does not provide sufficient information (e.g., the category may be too broad or reflect multifactorial disease), and studies utilizing more specific methods of outcome ascertainment would be useful for establishing coherence and biological plausibility for a link between PCB exposure and cause-specific mortality.

Exposure timing is an important consideration for both human and animal studies, especially for assessing the sensitivity of studies evaluating the potential for PCB effects on development. Many human studies measured PCBs in maternal blood, with assessment of other developmental exposure metrics also common, including breast milk, cord blood, and child blood. There were also >200 nonhuman mammalian studies with developmental exposure designs.

Although human exposure to PCBs can occur through multiple routes, including dietary intake, inhalation, and dermal contact (Weitekamp et al., 2021), studies in the nonhuman mammalian database used primarily oral exposures, with some injection exposures, and very few studies of inhalation or dermal exposure. As described in the introduction, PCB inhalation in contaminated buildings has been an area of great public health interest, especially in the context of PCB exposure in schools (Associated Press, 2022; Blatchhford, 2022; US EPA, 2019, 2015, 2012). The scarcity of PCB data for inhalation exposure represents one important area of uncertainty for risk assessment (US EPA, 2019; Lehmann et al., 2015; US EPA, 2015).

Whether the data are collected in humans or animals, databases with the potential to inform coherence across biologically related endpoints are more likely to support strong hazard conclusions than databases including evaluations of only single endpoints in isolation. While a change in a single endpoint or biomarker of effect could be due to random chance, changes in several related endpoints are less likely to occur solely for this reason. In some instances, changes in multiple endpoints can indicate greater severity or progression along a disease continuum or provide more insight into possible mechanisms/modes of action. For both humans and nonhuman mammals, potential impacts of PCB exposure were evaluated on a wide range of health endpoints across all 15 organs/systems described below; more comprehensive summaries of the databases for each organ/system can be found in (US EPA, 2023). In humans, the most studied organs/systems were reproductive, nervous system, and metabolic. In nonhuman mammals, the most studied organs/systems were hepatobiliary, reproductive, and endocrine. Additional interactive visualizations describing human and nonhuman mammalian health endpoints are available in the Health Assessment Workspace collaborative (https://hawcprd.epa.gov/summary/assessment/100500282/visuals/).

#### 3.2.2. Organ/system database summaries

Human studies evaluated a range of pathogenically related cardiovascular endpoints such as ischemic heart disease (IHD; also referred to as coronary artery disease or coronary heart disease); myocardial infarction (MI; heart attack); hypertension (high blood pressure); cerebrovascular disease; atherosclerosis (plaque buildup inside arteries and hardening and narrowing of their walls); and heart failure (HF; inability to pump sufficient blood to organs and tissues). The most common cardiovascular endpoints evaluated in studies of PCB exposed mammals other than humans included heart size/weight, cardiovascular histopathology, and blood pressure. However, an important challenge in experimental studies of cardiovascular endpoints is the resistance of wild-type rodents (specifically, mice and rats) to the development of vascular toxicity (Zhao et al., 2020b; Oppi et al., 2019). Piglets and monkeys are much better models of cardiovascular toxicity (Daugherty et al., 2017); however, these species have been underutilized in toxicology studies. In contrast to humans, rodents do not develop spontaneous IHD, MI, HF, or stroke. Because of these differences, drawing inferences about human cardiovascular health risk based on the results of rodent studies can be especially challenging. For example, although most experimental studies indicate no or minimal impact on heart weight and histopathology of the cardiovascular system upon chronic dietary exposure to PCB mixtures, this could result from the relative insensitivity of the most used mammalian models to the development of cardiovascular effects. Only recently, genetically altered mice lacking apolipoprotein-E or the low-density lipoprotein receptor have been introduced to evaluate cardiovascular impacts of PCBs. Although these studies did not administer PCB mixtures, they demonstrated accelerated atherosclerosis upon treatment with individual PCB congeners (Petriello et al., 2018; Murphy et al., 2015; Hennig et al., 2002). The results of these studies suggest a potential association between PCB exposure and atherosclerosis, especially in individuals prone to the development of vascular disease. The overall paucity of literature on this topic identifies it as an important area of uncertainty that would benefit from further research. Overall, the human database, supplemented with data from animal studies conducted using sensitive and informative models and methods, is likely to provide enough information to evaluate the potential for PCB-associated effects on the cardiovascular system. However, further study of PCB exposures and atherosclerosis and other cardiovascular endpoints in humans and in genetically altered mouse models of human vascular diseases is warranted to fully address the potential for PCB cardiovascular toxicity.

Responses of the skin and nails to chemical exposures can include dermal irritation and scar formation; and, specifically in the case of exposures to halogenated aromatic hydrocarbons, especially dioxin-like chemicals, acne and chloracne (Ju et al., 2009). Other endpoints evaluated in relation to PCB exposure include abnormal pigmentation, deformities in fingernails or toenails, hyperkeratosis, and gingival swelling or recession. Studies in mammals other than humans also evaluated alopecia, dermal/subcutaneous histopathological changes, and wound healing. Of the human studies, most were reports of occupational exposures, Yu-Cheng or Yusho poisoning, or other accidental exposures. In many of these studies, the dermal endpoints were symptoms reported or clinical observations after the exposure. Many dermal endpoints were part of the initial diagnosis and were not necessarily the focus of the publications. Although most human studies available for dermal endpoints were focused on populations with potential for relatively high exposure, two studies were conducted among general population samples, including one prospective birth cohort (Smit et al., 2015) and a population-based cross-sectional survey (National Health and Nutritional Examination Survey) focused on periodontal disease (Lee et al., 2008). The combined human and animal database is likely to provide enough information to draw conclusions about the potential for PCB exposure to cause dermal effects, especially at relatively high exposure levels.

Fetal development and early childhood can be particularly sensitive stages for adverse effects from exposure to environmental agents, as they are periods of rapid growth and development that can be affected by a range of toxicants through diverse biological mechanisms. Although exposures during development can impact any biological system, for the purpose of this review, developmental endpoints included in this domain are as follows: offspring mortality; body weight and size in early life, which includes fetal growth, anthropometric measures at birth, and childhood height or weight status and rates of growth; birth defects; placental weight/histopathology; and, in animals, timing of postnatal developmental milestones, such as eye opening and pinna detachment. Most human studies of developmental endpoints reported measures of birth weight or other aspects of fetal growth, and (mainly in animal studies) offspring viability. The strength of the database for assessing human evidence of relationships between PCB exposure and developmental effects is high for birth weight and other growth parameters that can be collected at birth. Animal studies of other developmental endpoints are often available and represent exposures in multiple species at a wide range of doses, routes, durations, and developmental timings; these could provide additional information useful for evaluating potential hazards of PCB exposure.

Endocrine responses evaluated in relation to PCB exposure include the following organs and hormones: thyroid, adrenal glands, pituitary gland, parathyroid (PTH) glands, vitamin D, insulin-like growth factor (IGF) and melatonin. For this review, measures of sex-steroid hormones were classified as reproductive, and measures of insulin were classified as metabolic. Thyroid and adrenal activity are regulated by hypothalamic and pituitary hormones through negative feedback pathways, comprising the hypothalamus-pituitary-thyroid (HPT) and hypothalamus-pituitary-adrenal (HPA) axes. Potential effects on the HPT axis include thyroid disease, changes in circulating thyroid hormone concentrations, and thyroid histopathology. A strong database of human studies for thyroid function is available, comprising multiple moderate to large prospective studies assessing occupational and nonoccupational PCB exposures, using valid endocrine biomarkers (i.e., thyroid hormone levels). The human studies are supported by extensive experimental data from nonhuman mammalian studies. Endocrine responses evaluated with PCB exposure also include differences in circulating adrenal hormone concentrations and histopathological adrenal changes. The strength of the human database for adrenal effects is limited by the few modest-sized (mostly cross-sectional) studies. In contrast, the nonhuman mammalian database is more robust and can provide information on potential links between PCB exposures and changes in levels of circulating glucocorticoids and structural alterations of the adrenal cortex. Other endocrine endpoints studied in nonhuman mammals included pituitary gland weight and histopathology, PTH structure and hormones, vitamin D, IGF, and melatonin. Very few human studies measured these endpoints. The lack of information for these other hormones precludes the ability to draw hazard conclusions and represents an area of uncertainty that would benefit from further research.

Studies of gastrointestinal (GI) endpoints evaluated GI histopathology or gut permeability (in nonhuman mammals), abdominal ultrasonography (in humans), and digestive system symptoms and diseases (in humans or other mammals), including specific clinical conditions (e.g., gastric ulcer and colorectal polyps) and more subjective symptoms such as abdominal pain, nausea/vomiting, changes in bowel habits, bloating, indigestion, and loss of appetite. Additional endpoints evaluated in nonhuman mammals included intestinal bleeding, bloody stools, edema, intestinal blockage, and diverticula of the large bowel. Human studies were relatively few and sometimes evaluated non-specific or subjective endpoints. For example, two human studies assessed mortality due to digestive system diseases; however, this endpoint is nonspecific and often includes liver disease. The most informative studies for assessing the potential for PCB exposure to cause GI effects are those that examined GI histopathology, abdominal ultrasonography, and specific clinical conditions. These were relatively rarely studied in humans with relation to PCB exposure; consequently, hazard identification for PCBs would likely depend on animal studies of GI histopathology to help understand changes in GI structure and function related to exposure.

There were numerous studies evaluating hematopoietic endpoints, including red blood cell, white blood cell (WBC), and platelet counts, as well as bone marrow histopathology (in nonhuman mammals), likely providing a sufficient foundation for evaluating hazard for these endpoints. However, there are very few studies for endpoints related to hemostasis (e.g., clotting function), which represents an area of uncertainty that would benefit from further research.

Studies of hepatobiliary endpoints evaluated metabolic enzymes, liver size/weight, liver histopathology or ultrasonography, liver lipid levels and steatosis, frank liver disease and cirrhosis, serum biomarkers of liver function, porphyrin levels, blood and liver levels of fatty acids, lipids and cholesterol, hepatic micronutrient content, bile acid content and excretion, and gall bladder histopathology. Eleven human studies included direct evaluations of liver injury; those consist mostly of prospective studies on cirrhosis mortality in occupational cohorts. Levels of serum biomarkers of liver function (e.g., alanine aminotransferase, aspartate aminotransferase, bilirubin, alkaline phosphatase, and gamma-glutamyltransferase) and of triglycerides and cholesterol were more frequently measured in human studies although the bulk of these studies were cross-sectional. Particularly in the case of lipid measurements, reverse causality whereby lipophilic PCBs accumulate more in individuals with higher lipid levels is a possibility, and cross-sectional studies are limited in that they do not establish temporality between the exposure and outcome. However, there is a robust database of nonhuman mammalian studies to establish temporality between PCB exposures and these endpoints at a wide range of exposure levels. Animal studies additionally reflect the potential for effects of PCB exposure on a broad range of structural and functional parameters of the liver, including histological evaluations of liver damage and evaluations of liver enzyme induction and liver weight changes. Therefore, the overall database likely provides sufficient information to draw hazard conclusions for effects of PCB exposure on the liver. However, we identified no human studies of gall bladder structure or function, and only four studies in nonhuman primates have investigated gallbladder and biliary duct histopathology. Bile acid content and excretion of bile were evaluated in nine studies of rats exposed to PCB mixtures. Additional prospective human studies of hepatobiliary endpoints, including more studies in general population cohorts, might strengthen the database, while gallbladder and biliary endpoints represent areas of uncertainty that would benefit from further research.

The immune system is highly dispersed, comprising multiple organs, tissues, and cell types, the main function of which is to ensure homeoregulatory maintenance by preventing or limiting infection and malignancy (IPCS, 2012). Adverse effects can result both from suppression of the immune system, as well as by inappropriate stimulation (e.g., allergy). For this review, endpoints included in this domain are as follows: susceptibility to infection/malignancy, atopy (i.e., allergy and asthma), autoimmune disease, antigen-specific antibody responses (e.g., to vaccination), WBC function, delayed-type hypersensitivity (DTH) responses, antibody levels, immune organ size and weight, immune organ histopathology, and endotoxin sensitivity. With respect to identifying hazards from PCB exposure, the most informative immune effects are those that indicate a change in immune function in response to challenge, such as susceptibility to infection, atopy, autoimmune disease, antigen-specific antibody responses, WBC function (e.g., lymphocyte proliferation assays, natural killer cell activity assays), and DTH (IPCS, 2012). Thirty-one human studies evaluated occurrence of infectious disease, although many studies relied on questionnaires of the participant (or parent) to ascertain outcomes. The human data can be supplemented with data from rodent studies of host resistance to infectious agents or tumor challenge. Response to immune challenge was evaluated in seven human studies (after vaccination) and 28 nonhuman mammalian studies. Forty-three human studies evaluated PCB exposure in relation to allergy or asthma. While only 3 human studies evaluated autoimmune disease, 23 measured autoantibody levels. Overall, sufficient information is available to draw hazard conclusions for immune effects and PCB exposure, especially with regard to immune suppression, with smaller amounts of data available for allergies/asthma and autoimmunity.

Studies of metabolic endpoints evaluated pathophysiologically related markers of impaired glucose metabolism and overweight/obesity. Impaired glucose metabolism was evaluated in relation to PCB exposure in humans based on the following endpoints: insulin resistance (IR); impaired glucose tolerance (IGT)/prediabetes; type 2 diabetes mellitus; gestational diabetes; diabetes mellitus (not otherwise specified); and metabolic syndrome. Many human studies evaluated informative endpoints such as IR, IGT, and type 2 and gestational diabetes, which can all be verified using standard clinical laboratory tests, although with varying accuracy depending on specific method. Metabolic endpoints were also evaluated in a substantial database of nonhuman mammalian studies, including endpoints not represented in the human database, such as pancreas weight and histopathology, adipose tissue histopathology, and basal metabolic rate. Together, the human and animal databases are likely to provide enough information to evaluate the potential for PCB-associated metabolic effects.

Musculoskeletal health endpoints, including bone strength and density, bone histopathology, bone development, dentition, skeletal muscle histopathology, muscle mass and tone, and arthritis were evaluated in human and experimental animal studies. Ten human studies evaluated subjective measures of musculoskeletal complaints and disease, while ten others evaluated bone strength and density and six evaluated dental abnormalities. Most human studies of bone density and strength or dental abnormalities were conducted among populations with potential for higher PCB exposure through regular consumption of fish or marine mammals, regional contamination from industrial sites, maternal occupation, or exposure via contaminated rice oil (Yu-Cheng population). Only three human studies evaluated PCB exposure and arthritis, which represents an area of uncertainty that would benefit from further research. Animal studies examined bone density, development, and histopathology as well as dental abnormalities and skeletal muscle histopathology. Across the human and animal literature, bone density and dental abnormalities were the most studied musculoskeletal endpoints, and the data for these might be sufficient to support an evaluation of musculoskeletal hazards of PCB exposure, especially at exposure levels higher relative to those seen in the general population.

Human studies of nervous system endpoints were conducted in a range of age groups from infants to older adults. For this review, we identified seven domains as most informative for assessing hazards of PCB exposure for the nervous system: cognitive function; attention, impulse control, externalizing behaviors, and internalizing behaviors (including activity level); executive function; social cognition and social behavior, including traits related to autism spectrum disorder (ASD); motor function/development; brain aging disorders; and auditory function. Note that these domains are not mutually exclusive, as they arise in an interconnected brain. For example, executive function bears greatly on cognitive processes involved in planning and working memory and is often impaired among individuals with attention deficit hyperactivity disorder and ASD. In addition to the human studies, there is also a substantial database of studies that examined nonhuman mammalian behaviors that have face validity to the seven most informative domains described above. Additional endpoint categories identified in eligible human studies outside these domains include olfactory function, visual function, neurological condition, peripheral sensation or pain, headache, dizziness, fatigue/level of consciousness, sleep problems, neurological disease mortality, neurophysiology or neuroimaging, and play behavior. However, these endpoint categories were considered less informative for hazard identification because they are broadly defined (e.g., neurological condition), provide a limited database (e.g., olfactory function, sleep problems, play behavior), or are inherently subjective (e.g., headache, fatigue). In addition, endpoints assessed within the neurophysiology/neuroimaging category (e.g., sensory and motor nerve conduction, nerve conduction velocity, event-related potentials, neuroimaging), as well as similar endpoints evaluated in nonhuman mammals (e.g., neuropathology and neurochemistry), are more mechanistic and better suited for a discussion of the mode-of-action evidence for PCB-induced neurological changes. Together, the human and animal databases provide sufficient information to evaluate the potential for PCB-associated nervous system effects, especially for cognitive function, attention, impulse control, externalizing and internalizing behaviors, executive function, and motor function. Numerous studies evaluating endpoints within these domains were identified that used well-accepted and validated endpoint measures with PCB exposures assessed across the lifespan.

Like chloracne, some effects on periocular tissues, such as Meibomian gland enlargement, ocular discharge, and periorbital edema, are markers of exposure to halogenated aromatic hydrocarbons, including PCBs (ATSDR, 2000). Other ocular endpoints evaluated in humans in relation to PCB exposure include ocular irritation, abnormal pigmentation of ocular tissues, and conjunctivitis. Most human studies of ocular endpoints were conducted following occupational or accidental exposures. These studies generally evaluated potential effects of higher exposures than would occur in the general population with a high likelihood of co-exposures to other chemicals. Many of the ocular endpoints studied were symptoms or clinical observations after the exposure and were not necessarily the focus of the publications. Nonhuman mammalian studies evaluated many of the same ocular endpoints included in human studies as well as histopathological changes in periocular tissues. Twenty-three of the nonhuman mammalian studies of ocular endpoints were conducted in primates; seven of these evaluated ocular histopathology. Overall, the database is likely to provide enough information to draw conclusions about the potential for PCB exposure to cause effects on periocular tissues, especially at higher exposure levels.

A wide range of reproductive endpoints was evaluated in both males and females; these endpoints are related via alterations in the synthesis, production, secretion or metabolism of sex-steroid hormones (estradiol, progesterone, and testosterone) and gonadotropins (follicle stimulating hormone and luteinizing hormone). Some endpoints may be affected by exposures in either or both members of a couple (e.g., time to pregnancy, fecundity, and fertility), while others are usually studied in only one sex. The most informative female reproductive endpoints in epidemiological studies are those that are clearly measurable, including age at menarche, menopause, presence of endometriosis, and hormone levels. Sexual maturation (including age at menarche in humans) was studied in a variety of human populations, as well as in nonhuman mammalian studies. Other female reproductive endpoints evaluated in multiple human and animal studies included endometriosis, menstrual irregularities, ovulation, and gestational length. Animal studies evaluated additional endpoints such as sexual behavior and histopathological evaluations of the uterus, ovaries, and vagina. In human adults, the most informative male reproductive endpoints are readily measurable sperm/semen parameters and reproductive hormones; these were also the most frequently reported male reproductive endpoints. Male reproductive organ size/weight was measured in only eight human studies but 72 nonhuman mammalian studies. Other male reproductive endpoints evaluated only in animal studies included sexual behaviors and histopathology. There were both human and animal studies that evaluated reproductive endpoints (such as time to pregnancy) in relation to exposure of both male and female partners. More animal studies evaluated the potential effects of maternal-only or paternal-only exposure on pregnancy or conception rate. Overall, the database of human and nonhuman mammalian studies is likely sufficient to support hazard identification for PCB exposure and reproductive endpoints.

Respiratory health in humans has been assessed through measurements of pulmonary structure (e.g., chest radiography), pulmonary function (e.g., lung volume and air flow), and respiratory symptoms (e.g., shortness of breath, cough, presence of sputum, and chest tightness). Many human studies were conducted in populations with occupational exposure, and the most commonly evaluated endpoints were respiratory disease mortality, pulmonary function, and respiratory symptoms. Respiratory endpoints assessed in nonhuman mammalian studies included pleural effusion, respiratory rate, blood gas tension, pulmonary histopathology, and lung weight. The most evaluated of these were pulmonary histopathology and lung weight. Overall, the human database is mostly limited to populations with high PCB exposure, some with simultaneous exposures to other compounds that could contribute to the effects observed. However, the combined human and animal database is likely to provide enough information to draw conclusions about the potential for PCB exposure to cause respiratory effects, especially at relatively high exposure levels.

In the kidney, pathophysiological responses to chemical exposures can range from subclinical elevations in urinary or serum levels of certain biomarkers to frank disease, including renal failure. Only four human studies evaluated frank renal disease or diabetic nephropathy; the majority instead measured biomarkers of renal function (e.g., serum levels of uric acid, urinary or serum creatinine, blood urea nitrogen, and urinary albumin), which were also measured in 28 nonhuman mammalian studies. Animal studies also evaluated endpoints such as kidney weight, histopathology and urine composition and output. Together, these human and animal databases are likely to provide enough information to draw conclusions about the potential for PCB exposure to cause effects on kidney structure and function. However, the urinary system also includes the urinary bladder. Urinary bladder histopathological changes have been evaluated in only eight nonhuman mammalian studies, five of which evaluated kidney and urinary bladder histopathology together (Arnold et al., 1997; Schaeffer et al., 1984; Chu et al., 1980; Iatropoulos et al., 1978; Koller and Zinkl, 1973). More data would be needed to support assessments of the effects of PCB exposure on the urinary bladder and potential mechanistic linkages among health endpoints evaluated across the full urinary system.

## 4. Conclusions

In this review, over 1500 studies of humans and other mammals exposed to PCB mixtures were mapped to 15 organs/systems. We identified 637 mammalian toxicological studies evaluating endpoints in a variety of species exposed for different durations and at different life stages and 953 epidemiological studies conducted using diverse populations and methods. Although human and animal data are abundant for some health endpoints (see Table 3^2^), other endpoints of great public interest (e.g., cardiovascular disease, autism) have not been extensively studied in the context of PCB mixture exposure.

Table 3 provides a high-level summary of the endpoints in each organ/system that were evaluated in human studies, along with preliminary assessments of the informativeness of each database that consider the availability of both human and other mammalian studies. More informative databases are more likely to support conclusive systematic reviews, while less informative databases could be used to identify important topics for future research. Endpoints identified as having low specificity might be most useful when evaluated in combination with other, more informative measures. The anthropocentric focus of Table 3 was chosen for simplicity and because endpoints grounded in strong human and animal data provide the most informative basis for hazard identification. However, endpoints evaluated only in animals can and often do provide sufficient evidence to support risk assessment (US EPA, 2020). Notably, sparse data exist for inhalation and dermal exposures, representing areas of uncertainty that would benefit from further research, especially because these are highly relevant human exposure routes.

There are several considerations that are important for interpreting this work. First, it relies on publicly accessible published data. Supplemental search strategies (e.g., searching reference lists of reviews, citation mapping, gray literature searches) were not extensively applied, but technical experts did identify a few additional references. Potential selective reporting of results is a factor to consider at the study evaluation step of a full systematic review. Furthermore, our analysis of the PCB database included only published reports and did not address the potential for publication bias. Future systematic reviews stemming from this work could consider using statistical approaches to assess publication bias for studies of health endpoints and exposure to PCB mixtures (Dalton et al., 2016). Second, this evidence map is based only on full-text screening and preliminary study extraction. Future analyses could include study evaluation and full data extraction of studies relevant for specific research questions. These additional review steps are needed to develop strong conclusions linking PCB exposure with health effects.

The primary goal of this evidence map was to use systematic review methods to identify, summarize, organize, and disseminate data relevant for characterizing potential human health concerns from exposure to PCB mixtures. As an important part of this effort, we developed interactive figures to help readers explore the available literature, including the ability to create lists of references with customized combinations of study design features based on the specific interests of the individual user. By sharing information from this systematic evidence map in this way, we hope to provide a valuable tool that will both support future risk assessment work and highlight areas of uncertainty (examples provided in Table 3) that can be prioritized in future research to advance our understanding of PCB mixtures and their potential effects on health.

## Supplementary Material

s1

s2

## Figures and Tables

**Table 1:** Populations, exposures, comparators, outcomes (PECO) criteria.

PECO element	Description of Studies Included

Populations	**Human**: Adults and/or children with exposure to PCBs at any life stage. **Animal**: Nonhuman mammalian animal species (whole organism) exposed during any life stage (during any period from in utero through adulthood). Studies including evaluations of transgenic animals only (i.e., with no evaluations of exposure-response relationships in wild-type animals) were considered “potentially relevant supplemental material.”
Exposures	**Human**: Any exposure to PCBs (in vivo) as determined by controlled exposure, measured PCB concentration in contact medium (e.g., food, air, dust), biomarkers of exposure (e.g., serum PCB levels), or occupation in a job involving exposure to PCBs (e.g., electric capacitor manufacturing). The following exposure assessment methods/exposure contexts were considered “potentially relevant supplemental material” in the absence of biomarker measurements or estimates derived using scientifically sound methods: Yusho/Yu-Cheng patient status; consumption of fish (or marine mammals or other wildlife); and residential proximity to a PCB-contaminated site. **Animal**: One or more oral (gavage, diet, drinking water, intragastric), inhalation (aerosol, vapor, or particle; whole-body or nose-only), dermal (occlusive, semiocclusive, nonocclusive), or injected (intravenous, subcutaneous, intraperitoneal) treatment(s) with any clearly quantified dosage of PCBs alone administered to a whole animal (in vivo).
Comparators	**Human**: A referent or comparison population that is unexposed or exposed at lower levels of PCBs, or exposed to PCBs for shorter periods of time, or cases versus controls, or a repeated measures design. However, worker surveillance studies are considered to meet PECO criteria even if no statistical analysis using a referent group is presented. Case reports and case series were considered “potentially relevant supplemental material.” **Animal**: A concurrent control group exposed to vehicle-only treatment or untreated control.
Outcomes	**Human**: Any examination of survival, body weight, or development, or of the structure or function of dermatological, cardiovascular, endocrine, gastrointestinal, hematological, hepatobiliary, immune, nervous, ocular, musculoskeletal, urinary, respiratory or reproductive cells, tissues, or systems. **Animal**: Any examination of survival, body weight, or development, or of the structure or function of dermatological, cardiovascular, endocrine, gastrointestinal, hematological, hepatobiliary, immune, nervous, ocular, musculoskeletal, urinary, respiratory or reproductive cells, tissues, or systems. *In general, endpoints related to clinical diagnostic criteria, disease outcomes, histopathological examination, or other apical/phenotypic outcomes are considered to meet PECO criteria and are prioritized for evidence synthesis while outcomes such as changes in cellular structure, gene expression, cell signaling, or other similar biochemical measures are considered “potentially relevant supplemental material. ”*

**Table 2:** Electronic prioritization of literature for hazard identification.

Group of studies	Prioritization approach	Number of prioritized studies

**2015 literature search (original search): 50,309 records retrieved – DoCTER Prioritization**		
Groups A–D (Fig. 1C)	DoCTER – Supervised Clustering	4605
Groups E–F (Fig. 1C)	DoCTER – Supervised Clustering and ML	3363
Studies with titles only	DoCTER – ML	3209
Total number electronically prioritized – DoCTER		**11,177**
**2017–2021 literature search updates**		
2017	SWIFT-Active Screener – Active	609
2018	ML	819
2019		855
2020		917
2021		842
Total number electronically prioritized – Active ML		**4042**

**Table 3:** Overview of the databases available for selected endpoint categories by organ/system.

Organ/system	Potentially more informative endpoint categories	Potentially less informative endpoint categories	Notes

Cardiovascular	Blood pressure/Hypertension IHD (including myocardial infarction) Cerebrovascular disease (including stroke)	*More data needed:* Atherosclerosis and other vascular diseases Heart failure Fetal heart rate *Low specificity or sensitivity:* Cardiovascular disease (NOS) Subjective complaints	Cholesterol/triglyceride levels were classified as hepatobiliary endpoints
Dermal	Acne/Chloracne Abnormal pigmentation Dermal irritation (including eczema) Hyperkeratosis Nail deformities	*More data needed:* Periodontal disease (including gingival swelling/recession) Scar formation *Low specificity or sensitivity:* “Skin problems”	Most human studies evaluated endpoints at high PCB exposure levels Atopic eczema was classified as an immune endpoint; dental abnormalities were classified as musculoskeletal endpoints
Developmental	Weight and size (early life)	*More data needed:* Miscarriage/stillbirth Birth defects *Low specificity or sensitivity:* Placental weight/histology	Measures of gestation length, pubertal development, and endpoints associated with testicular dysgenesis syndrome were classified as reproductive endpoints
Endocrine	Thyroid function	*More data needed:* Thyroid disease Adrenal gland function Potential effects on other endocrine organs and hormones (including parathyroid endpoints)	Sex hormone levels were classified as reproductive endpoints; insulin levels were classified as metabolic endpoints
Gastrointestinal	(none)	*More data needed:* Gastric ulcer Colorectal polyps Abdominal ultrasonography *Low specificity or sensitivity:* Abdominal pain Nausea/vomiting Changes in bowel habits Bloating Indigestion Loss of appetite	Database limited for hazard assessment
Hematopoietic	WBC counts Red blood cell counts/Hemoglobin	*More data needed:* Anemia Clotting function Platelet counts *Low specificity or sensitivity:* Blood disease mortality	Measures of WBC function were classified as immune endpoints
Hepatobiliary	Liver disease (including cirrhosis and steatosis) Serum biomarkers of liver function Cholesterol/Triglyceride levels Liver enzyme induction	*More data needed:* Porphyria Gallbladder and biliary endpoints *Low specificity or sensitivity:* Hepatomegaly	
Immune	Immune suppression (including susceptibility to infection, antigen-specific antibody responses, WBC function, DTH) Atopy (including allergies/asthma)	*More data needed:* Autoimmunity (especially autoimmune disease incidence) *Low specificity or sensitivity:* Nonspecific immunoglobulin levels WBC counts Immune organ size/weight	WBC counts were classified as hematopoietic endpoints
Metabolic	Glucose homeostasis (including IR, IGT/prediabetes, type 2 diabetes mellitus, gestational diabetes)	*More data needed:* Metabolic rate *Low specificity or sensitivity:* Metabolic syndrome Diabetes mellitus (NOS) Overweight/obesity	Cholesterol/triglyceride levels were classified as hepatobiliary endpoints; type 1 diabetes was classified as an immune endpoint; diabetic nephropathy was classified as a urinary system endpoint
Musculoskeletal	Bone density/strength Dental abnormalities (including enamel defects and dental caries)	*More data needed:* Arthritis *Low specificity or sensitivity:* Musculoskeletal complaints (including muscle/joint pain, muscle weakness)	Most human studies evaluated endpoints at high PCB exposure levels, especially in fish-consuming populations Rheumatoid arthritis was also classified as an immune endpoint
Nervous System	Cognitive function Attention, impulse control, externalizing and internalizing behaviors Executive function Motor function/development *Following developmental exposures:* Social cognition and behavior Auditory function	*More data needed:* Brain aging disorders Visual function Olfactory function Neurophysiology/neuroimaging *Following exposures during adulthood:* Social cognition and behavior Auditory function *Low specificity or sensitivity:* Dizziness Headache Fatigue/level of consciousness Neurological condition Neurological disease mortality Peripheral sensation or pain Play behavior Sleep problems	
Ocular	Ocular swelling and irritation (including periorbital edema, ocular discharge, Meibomian gland enlargement, conjunctivitis)		Most human studies evaluated endpoints at high PCB exposure levels Infectious forms of conjunctivitis were classified as immune endpoints
Reproductive	Sex hormone levels Fertility Sperm/semen parameters Gestation length (including preterm birth) Endometriosis Pubertal development Endpoints associated with testicular dysgenesis syndrome (including anogenital distance, hypospadias, cryptorchidism, sex ratio)	*More data needed:* Gynecological disorders other than endometriosis Pregnancy-related disorders (including preeclampsia) Menstrual cycle characteristics Ovulation Reproductive aging *Low specificity or sensitivity:* Erectile dysfunction	Measures of birth defects, miscarriage/stillbirth, and placental health were classified as developmental endpoints
Respiratory	Pulmonary health and function (including chest radiography, spirometry, respiratory sounds, sputum analysis)	*Low specificity or sensitivity:* Respiratory disease mortality Respiratory illness history Respiratory symptoms (e.g., shortness of breath, cough/sputum, chest tightness)	Most human studies evaluated endpoints at high PCB exposure levels Asthma and infectious respiratory diseases were classified as immune endpoints
Urinary System	Biomarkers of renal function (including markers measured in serum or urine)	*More data needed:* Kidney disease/renal failure (including diabetic nephropathy) Potential effects on urinary system components other than the kidneys *Low specificity or sensitivity:* Urinary system mortality (NOS)	

Note: DTH = delayed type hypersensitivity IGT = impaired glucose tolerance; IR = insulin resistance; IHD = ischemic heart disease; NOS = not otherwise specified; PCB = polychlorinated biphenyl; WBC = white blood cell.

## Data Availability

No data was used for the research described in the article.

## Footnotes

2 This table provides an overview of the available data relevant for evaluating select endpoints within each organ/system, with a focus on endpoints evaluated in human studies. Preliminary assessments of the potential informativeness of each database are included, with availability of evidence from nonhuman mammalian studies contributing to those assessments. Endpoints measured only in nonhuman mammalian models are not included in this table to reduce the complexity of the analysis and to focus on the endpoints with the most direct human relevance.

## Abbreviations:

PCB: polychlorinated biphenyl
SR: systematic review
SEM: systematic evidence map
HHRA: human health risk assessment
