# Supplementary material for: A systematic evidence map for the evaluation of noncancer health effects and exposures to polychlorinated biphenyl mixtures
Source: Environ Res. Author manuscript; Available in PMC 2024 Mar 1. (PMC10013199; doi:10.1016/j.envres.2022.115148)
Supplement: s1 [file NIHMS1875284-supplement-s1.docx]

**Modified PRISMA Report**

**Title of submitted paper and corresponding author**: A Systematic Evidence Map for the Evaluation of Noncancer Health Effects and Exposures to Polychlorinated Biphenyl Mixtures. Geniece M. Lehmann ([lehmann.geniece@epa.gov](mailto:lehmann.geniece@epa.gov))

| **#** | **Item** | **Guidance** | **Section Information is Located In** | **Manuscript Quote / Author Comments** |
| --- | --- | --- | --- | --- |
| **Title** | | | | |
| 1 | Title | Identify the report as a systematic map. | Title, Abstract | The manuscript title indicates the report is a systematic map (*Systematic Evidence Map for Noncancer Health Effects and Exposures to Polychlorinated Biphenyl (PCB) Mixtures*). The abstract and introduction also mention the report is a systematic map. |
| **Abstract** | | | | |
| 2 | Structured summary | Provide a structured summary including, as applicable:   - Background; - Objectives; - Data sources; - Study eligibility criteria; - Study appraisal methods, if conducted; - Results; - Limitations; conclusions and implications of key findings; - Systematic map registration number. | 1 (Introduction); 2 (Methods) | In the summary provided in the abstract and in the methods section, we provide the background, objectives, data sources, and study eligibility criteria. The protocol registration information from 2019 via Zenodo (<https://doi.org/10.5281/zenodo.3585771>) which is in the Methods section. Study appraisals were not conducted and are not a part of this evidence map. The results are reported in Section 3. Limitations, conclusions, and implications of findings are discussed in Section 4. |
| **Introduction** | | | | |
| 3 | Rationale | Describe the rationale for the map. | 1 (Introduction) | In the introduction, we note that “New research in the years since ATSDR’s assessments were published has suggested the potential for additional hazards, notably metabolic and cardiovascular effects. Furthermore, the full database of PCB literature has never been reviewed using systematic methods with the intent to identify most of the available evidence informing noncancer health hazards of exposure to PCB mixtures” |
| 4 | Objectives | Define primary and secondary questions for the systematic map. | 1 (Introduction) | In the introduction, we provide objectives for the map “This evidence map’s main objective is to summarize available noncancer health outcome data for mammalian toxicology and human epidemiology studies of exposures to PCB mixtures. By identifying health outcomes with evidence bases sufficient to support evaluations of coherence across both human and animal evidence and biologically related endpoints, we are able to highlight outcomes for future systematic reviews that have the highest likelihood of supporting a causal analysis of relationships with exposure to PCB mixtures. In addition, data gaps can be identified to inform future research efforts. Conversely, by identifying areas with poorer databases, this evidence map can also be used to inform future research efforts on topics that have been insufficiently studied.” |
| **Methods** | | | | |
| 5 | Protocol and registration | Indicate if a map protocol exists, if and where it can be accessed (e.g., Web address), and, if available, provide registration information including registration number. | 2 (Methods) | The systematic review protocol was published in 2019 and is accessible here:  <https://zenodo.org/record/3585772#.Xfu_okdKhaR>. |
| 6 | Eligibility criteria | Specify characteristics of study reports used as criteria for eligibility, giving rationale. | 2 (Methods) | PECO criteria for study eligibility are included in Table 1 and described in the Methods Section 2. |
| 7 | Information sources | Describe all information sources (e.g., databases with dates of coverage, contact with study authors to identify additional studies) in the search and date last searched. | 2 (Methods) | The literature search is described in the Methods section “The literature search strategy relied on terms describing PCB mixtures and individual congeners (e.g., “polychlorinated biphenyls”, “Aroclor”, “PCB”, “tetrachlorobiphenyl”) to gather information on exposure to the chemicals of interest. Additional exposure terms were used to identify studies that were not indexed by the chemical name (e.g., "capacitor manufacturing workers", "Yu-Cheng", "New York State Angler Cohort"). These search terms were intentionally broad and did not prioritize studies in which exposure was quantified; this was considered during screening of the literature. The detailed search strategies are presented in Supplemental File 1. Peer-reviewed literature was identified by searching PubMed (National Library of Medicine), Web of Science (Clarivate Analytics), and Toxline (National Library of Medicine). The original search, conducted in 2015, was not restricted by publication date or language. Literature search updates were conducted yearly and were restricted to the 12-month period since the original search or the most recent update. Records identified through the original literature search were prioritized electronically as described below.” The literature was updated to include all the 2021 literature just prior to submission. |
| 8 | Search | Present full electronic search strategy for at least one database, including any limits used, such that it could be repeated. | Supplemental file 1 | The detailed search strategies are presented in Supplemental File 1. |
| 9 | Study selection | State the process for selecting studies (i.e., screening, eligibility, included in systematic map). | 2 (Methods) | PECO criteria are included in Table 1. Screening (title / abstract and full text) are described in the methods sections 2.1 through 2.3. All studies that were identified as PECO relevant were included in the systematic map. |
| 10 | Data collection process | Describe method of data extraction from reports (e.g., piloted forms, independently, in duplicate) and any processes for obtaining and confirming data from investigators. | 2 (Methods) | References retrieved through August 2016 were screened and tagged using DRAGON. Screening decisions and study metadata recorded in DRAGON (v. 03-25-2016) were recently moved to a second generation, web-based platform rebranded as litstream^TM^ (ICF, 2019). References identified in search updates after August 2016 were screened in SWIFT-Active Screener until the software indicated a likelihood of 95% that all relevant studies had been captured. This threshold is comparable to human error rates (Bannach-Brown et al., 2018; Howard et al., 2016; Cohen et al., 2006) and is used as a metric to evaluate ML performance. A summary of literature prioritized using SWIFT-Active Screener is provided in Table S1F. These structured forms were used for screening and Excel forms were used for inventory development mentioned in methods .“On the basis of the results for the full-text review, summary-level, sortable lists of relevant literature were created for human and animal (nonhuman mammalian) studies for each health system. Fundamental study design information (e.g., study population, exposure assessment/design, health endpoints evaluated) was extracted for each study in Microsoft Excel by one individual and independently reviewed by at least one additional individual.” |
| 11 | Data coding strategy | List and define all variables for which data were sought and any assumptions and simplifications made. | 2 (Methods) | In the methods, we describe that studies were tagged to specific health effects, including “Cardiovascular, Dermal, Ocular, Developmental, Endocrine, Gastrointestinal, Hematopoietic, Hepatobiliary, Immune System, Metabolic Disease, Musculoskeletal, Nervous System, Ocular, Reproductive, Respiratory, and Urinary System.” These health effect categories were chosen because of their potential to include specific noncancer health outcomes responsive to PCB exposure at the lowest levels, which would be of the most interest for public health. Studies focused entirely on health effect data outside of these categories, including effects on mortality of unknown cause, wasting, and cancer were considered potentially relevant supporting supplemental studies. We also note that further study information was captured in excel based inventories, “Based on the results of the full-text review, separate human and mammalian literature inventories were created for each health effect category to develop summary-level, sortable lists of relevant literature. Fundamental study design information (e.g., study population, exposure assessment/design, health endpoints evaluated) was extracted for each study in Microsoft Excel” |
| 12 | Study quality assessment | If conducted, describe methods for assessing quality of individual studies. |  | N/A (Study quality assessment not conducted in this evidence map) |
| **Results** | | | | |
| 13 | Study selection | Give numbers of studies screened, assessed for eligibility, and included in the review, with reasons for exclusions at each stage, illustrated with a PRISMA flow diagram. | 3 (Results and Discussion); Figure 3 | The results of the study selection are described in results (Section 3.1), and in the health effect summaries (Section 3.2). The literature flow diagram (PRISMA flow diagram) is included as Figure 3 which displays the information on number of studies screened, included, and excluded at each step in the review. The raw counts are available as Ris Endnote files in supplement, and the supplemental file 1J includes the exact study information used to generate the interactive figures. |
| 14 | Study database | Show how the relevant literature is organised (categories, coding etc.) according to transparent, replicable criteria. This map should be readily updateable. | HAWC Tableau visualizations; Figures 4-10 | Interactive heat map visualizations are provided as Tableau figures in HAWC <https://hawc.epa.gov/summary/assessment/100500282/visuals/>. Static versions of each interactive dashboard are provided as Figures 4 to Figure 10. All data is downloadable and interactive through the visualizations. |
| 15 | Study quality assessment | Present map of study quality and preliminary estimate of the quality of the evidence base. |  | N/A (Study quality assessment not conducted in this evidence map) |
| **Discussion** | | | | |
| 16 | Summary of evidence | Observe distribution of articles, relative quantity, quality etc. as relevant to map objectives. | 3 (Results and Discussion) | In Section 3.2, overview health effect summaries are provided for human and animal evidence and brief summaries are provided for each of the health effect categories (Cardiovascular, Dermal, Ocular, Developmental, Endocrine, Gastrointestinal, Hematopoietic, Hepatobiliary, Immune System, Metabolic Disease, Musculoskeletal, Nervous System, Ocular, Reproductive, Respiratory, and Urinary System)  Interactive heat map visualizations are provided in HAWC that provide specific study number information and allow users to generate custom reference lists based on specified sorting https://hawc.epa.gov/summary/assessment/100500282/visuals/. |
| 17 | Limitations | Describe limitations of the map, such as limitations in search strategy, potential bias in pool of retrieved articles, etc. | 4 (Conclusions) | Limitations of the search strategy are outlined in the methods (Section 2.1) and conclusions (Section 4), noting that our literature search strategy did not include supplemental search strategies (searching reference lists of reviews, citation mapping, grey literature searches, etc). In the conclusions, we note that “a limitation of this work is that it relies on publicly accessible published data. Historically, scientific journals have often enforced publication length restrictions, sometimes resulting in incomplete reporting of study results. Potential selective reporting, where authors fail to mention they conducted an evaluation or fail to report the results of an evaluation, is a factor to consider at the study evaluation step of a full systematic review. Furthermore, there has also been some reluctance by journals to publish reports with primarily null results {Horvatt 2017, 29180912} and possibly some work that has not been published based on decisions made by study sponsors or other factors {Bero, 2016, 7607845}. Publication bias exists when the reasons behind a failure to publish are associated with the direction or magnitude of the effects observed {Dwan, 2008, 18769481; Dwan, 2013, 23861749}. Our analysis of the PCB database included only published reports and did not address the potential for publication bias. Future systematic reviews stemming from this work could consider using statistical approaches to assess publication bias for studies of health outcomes and exposure to PCB mixtures {Dalton, 2016, 7607849}.“ |
| 18 | Conclusions | Describe both policy and management implications, and implications for research. | 3 (Results and Discussion), 4 (Conclusions) | The research implications are discussed in each health effect summary for every category in Section 3.2, but in the conclusions (Section 4) we also note “The primary goal of this evidence map was to use systematic review methods to identify, summarize, organize, and disseminate evidence relevant for characterizing potential human health concerns from exposure to PCB mixtures. As an important part of this effort, we developed interactive figures to help readers explore the available literature, including the ability to create lists of references with customized combinations of study design features based on the specific interests of the individual user. By sharing information from this systematic evidence map in this way, we hope to provide a valuable tool that will both support future risk assessment work and highlight data gaps that can be prioritized in future research to advance our understanding of PCB mixtures and their potential effects on health.” |
| **Funding** | | | | |
| 19 | Funding | Describe sources of funding for the map, other support, and role of funders. | NA | PJL declares she is a paid consultant for Friedman Rubin Associates (Seattle, WA) representing parents of children who attend Sky Valley School in Monroe, WA in the case of Sky Valley v. Monsanto. CRK discloses that she is currently employed by and receives a salary from Bausch Health Companies, Incorporated. CRK also owns shares in Bausch Health Companies Stock and retirement investments. Remaining authors declare no financial conflict of interest. This material has been funded in part by the US Environmental Protection Agency under Contract 68HERC19D0003 to ICF. Contributions from the following co-authors were supported by the US Environmental Protection Agency under Contract 68HERC19D0003 to ICF: SKS, PR, CRK, PJL, PFL, AS, MT, MSB, JT, TAJ, LR, JM, AFK, RB, RS, SS, CL, KS, and BI. The views expressed are those of the authors and do not necessarily represent the views or policies of the US Environmental Protection Agency. Any mention of trade names, products, or services does not imply an endorsement by the US Government or the US Environmental Protection Agency. EPA does not endorse any commercial products, services, or enterprises. |
